# Sequence-Based Analysis Uncovers an Abundance of Non-Coding RNA in the Total Transcriptome of *Mycobacterium tuberculosis*

**DOI:** 10.1371/journal.ppat.1002342

**Published:** 2011-11-03

**Authors:** Kristine B. Arnvig, Iñaki Comas, Nicholas R. Thomson, Joanna Houghton, Helena I. Boshoff, Nicholas J. Croucher, Graham Rose, Timothy T. Perkins, Julian Parkhill, Gordon Dougan, Douglas B. Young

**Affiliations:** 1 Division of Mycobacterial Research, MRC National Institute for Medical Research, London, United Kingdom; 2 The Wellcome Trust Sanger Institute, Wellcome Trust Genome Campus, Cambridge, United Kingdom; 3 NIAID, National Institutes of Health, Bethesda, Maryland, United States of America; Johns Hopkins School of Medicine, United States of America

## Abstract

RNA sequencing provides a new perspective on the genome of *Mycobacterium tuberculosis* by revealing an extensive presence of non-coding RNA, including long 5’ and 3’ untranslated regions, antisense transcripts, and intergenic small RNA (sRNA) molecules. More than a quarter of all sequence reads mapping outside of ribosomal RNA genes represent non-coding RNA, and the density of reads mapping to intergenic regions was more than two-fold higher than that mapping to annotated coding sequences. Selected sRNAs were found at increased abundance in stationary phase cultures and accumulated to remarkably high levels in the lungs of chronically infected mice, indicating a potential contribution to pathogenesis. The ability of tubercle bacilli to adapt to changing environments within the host is critical to their ability to cause disease and to persist during drug treatment; it is likely that novel post-transcriptional regulatory networks will play an important role in these adaptive responses.

## Author Summary

Tuberculosis bacteria are able to hide quietly inside the body for years or decades before reawakening to cause disease. If we knew more about how the bacteria change from a harmless persistent form to an aggressive disease-causing form, we could develop drugs that would be more effective in treating active tuberculosis and may also allow us to eliminate the infection before it erupts into disease. The key to this is in knowing how the bacteria determine which of their genes to express at different times. By applying modern sequencing technologies we have discovered a new putative network of gene regulation in *Mycobacterium tuberculosis* that is based on RNA molecules rather than protein molecules. We anticipate that this finding will open the way for new research that will allow us to understand the fundamental mechanisms underlying this deadly human disease, and that will help us to design better tools for prevention and treatment of TB.

## Introduction

*Mycobacterium tuberculosis* presents a major threat to global health, causing around 10 million new cases of tuberculosis and 2 million deaths every year [1]. The bacteria typically establish a prolonged asymptomatic infection that progresses to active disease in only a minority of individuals. During the course of infection, *M. tuberculosis* has to adapt to survival in a range of different microenvironments, combining persistence in a non-replicating state with periods of active cell division [2]. Transcriptional regulation in response to environmental change has been extensively analyzed in *M. tuberculosis* by RT-PCR and hybridization to microarrays representing the complete set of predicted coding sequences (CDSs) [3], and several regulons induced under physiologically relevant growth conditions have been characterized [4]–[9]. Thirteen sigma factors, eleven two-component regulators, eleven serine-threonine protein kinases and more than one hundred predicted transcription factors have been identified in the genome of *M. tuberculosis* [10].

Less attention has been given to the potential role in *M. tuberculosis* of regulatory processes that occur subsequent to the initiation of transcription. These are often mediated by RNA and involve alterations in the efficiency of transcription, translation and stability of messenger RNAs (reviewed in [11]–[13]). There are two broad categories of non-coding regulatory RNA [11]–[13]. The first is based on *cis*-acting regulatory elements that are present on the mRNA transcript, generally as a 5’ untranslated region (UTR). 5’ UTRs can regulate gene expression by forming secondary structural features that enhance or inhibit transcription or translation of their cognate mRNA. Their structural conformation is often determined by interaction with particular protein, tRNA or small molecule ligands. 5’ UTRs that respond to small molecules are referred to as riboswitches [14]. The second category of RNA regulators is small RNA molecules (sRNAs) that bind in *trans* to their mRNA target. These include antisense RNAs that are transcribed from the DNA strand opposite to a CDS, inhibiting translation and promoting degradation by base-pairing with the corresponding mRNA transcript [13]. A second class of sRNAs are transcribed from a separate location on the genome – typically an intergenic region (IGR), though attenuated 5’ UTR transcripts can also act as sRNAs [15] – and again influence translation and degradation of mRNAs by a more limited degree of base pairing. These “*trans*-encoded” sRNAs are functionally analogous to microRNAs in eukaryotic cells [16].

RNA regulators are frequently implicated in adaptive responses of bacteria to environmental change and may therefore be expected to play a role in the pathogenesis of *M. tuberculosis*. sRNAs have been shown to regulate expression of virulence determinants in a number of bacterial pathogens, located in some cases within defined pathogenicity islands and carried specifically by pathogenic strains [17]–[20] (reviewed in [21]). Initial studies have described the occurrence of sRNAs in *M. tuberculosis* though the extent of the regulatory RNA network in these bacteria, and its relevance to gene expression during infection, are unknown [22], [23].

Knowledge of regulatory networks controlled by non-coding RNA in bacteria has undergone a rapid expansion over the last decade, as a result of transcriptional profiling approaches based on high-density tiling arrays and deep sequencing of whole genome cDNAs (RNA-seq). Sequence-based transcriptomes have been described for a range of bacteria, including several human pathogens [17], [24]–[29]. The aim of the present study was to apply an analogous approach to characterize the transcriptome of *M. tuberculosis*, with a particular focus on identification of the nature and extent of non-coding RNA.

## Results

### The total transcriptome of *M. tuberculosis*

RNA extracted from three independent exponential phase cultures of *M. tuberculosis* was used to generate cDNA preparations that were then analyzed by Illumina-based sequencing. Two of the samples were analyzed as technical replicates in separate sequencing runs, generating a set of five total transcriptome profiles (Table 1). After removal of reads mapping to rRNA molecules an average of just over 3 million reads, corresponding to 8% of total, mapped to annotated protein CDSs in the sense orientation. A further 0.5 million reads mapped to CDSs in the antisense orientation, and 0.7 million reads mapped to IGRs. IGRs make up less than 10% of the genome, and thus the density of reads mapping outside of CDSs was more than two-fold higher than that seen for predicted coding transcripts. Calculation of pairwise correlation coefficients demonstrated a high degree of reproducibility between samples (r≥0.93 Table S1), and the total transcriptome data are recorded as an average of the five samples in Table S2 (sense and antisense reads for each CDS) and Table S3 (reads mapping to IGRs of at least 50 nucleotides). To facilitate comparison of expression levels of different genes, data for CDSs are also presented in the form of reads per kilobase per million reads (RPKM).

**Table 1:** RNA sequencing profiles for exponential phase cultures of *M. tuberculosis.*

sample	4347_8	4349_2	4349_6 (t)	4349_3	4349_5 (t)	average	% of total	% of CDS
total mapped reads	34	40	31	45	45	39	-	-
reads mapped to CDS	2.8	3.3	3.3	2.8	2.9	3.1	8.0	-
reads antisense to CDS	0.46	0.56	0.51	0.69	0.30	0.51	1.3	16.1
reads mapped to IGRs	0.97	0.79	0.62	0.80	0.51	0.74	1.9	23.4

Values are shown as million reads; (t) technical replicate.

cDNA was also sequenced from two stationary phase cultures of *M. tuberculosis*. The correlation coefficient between the samples (r = 0.82) indicated a lower level of reproducibility than that seen in exponential phase. This was driven mainly by a lower amount of mRNA, with only 2% of stationary phase reads mapping to CDSs as compared to 8% in exponential phase as well as a dramatic increase in the level of intergenic reads (Table 2). However, the overall pattern of relative gene expression was very similar between the replicate samples, and data are expressed as average values in Tables S2 and S3.

**Table 2:** RNA sequencing profiles for stationary phase cultures of *M. tuberculosis.*

sample	4349_7	4349_8	average	% of total	% of CDS
total mapped reads	47	33	40	-	-
reads mapped to CDS	1.3	0.22	0.76	1.9	-
reads antisense to CDS	0.33	0.09	0.20	0.5	27.0
reads mapped to IGRs	1.8	0.81	1.3	3.3	175.0

Values are shown as million reads.

### The coding transcriptome

#### Exponential phase

**Figure 1:**
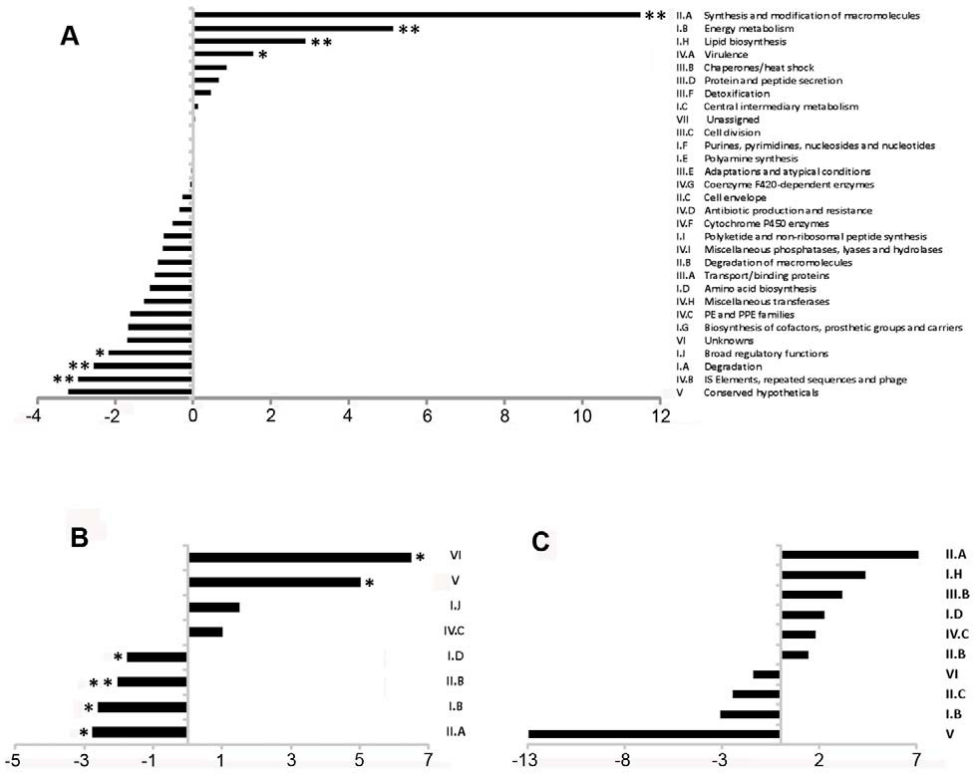
Representation of functional classes in the *M. tuberculosis* transcriptome. Transcripts identified by sequence analysis were grouped according to the functional class of their predicted gene product as assigned in the original genome annotation [10]. For all panels, values on the x-axis represents a difference in percentage, positive values indicate over-representation of a particular functional class whereas negative values indicate under-representation. Panel A shows the difference in the percentage of the functional classes among the 10% highest expressed genes of the transcriptome (N = 400) when compare to the percentage observed in the annotated genome. Panel B shows the difference in percentage of selected functional classes when comparing the transcripts with abundant antisense levels (antisense to sense ratio >0.5, N = 435) with the total set of CDSs with RPKM ≥5 (N = 3,136). Panel C shows the difference in percentage for selected functional classes when comparing the transcripts with abundant 5’ UTRs (see text for details, N = 82) with the total set of CDSs with RPKM ≥5 (N = 3,136). For B and C, no significant differences in representation were observed for functional classes that are not shown. * p<0.05 using Fisher's exact test. ** significant after multiple test correction (False Discovery Rate method).

To define the coding transcriptome, we selected CDSs with RPKM ≥5 in either sense or antisense direction. This included 3,136 CDSs during exponential culture (Table S2), representing 78.4% of the annotated genome. Table 3 shows the fifty CDSs with highest RPKM scores. To test for an association between expression level and gene function, we selected the 10% of CDSs with highest RPKM and compared the frequency with which different functional classes were represented in this selected set against the corresponding frequency in the total genome [10] (Figure 1A). Analysis by functional class shows a profile consistent with that expected for actively growing bacteria, with an over-representation of mRNA transcripts encoding proteins involved in the generation of energy and macromolecules (classes II.A, I.B, I.H, P−value<0.001 for all cases). Under-represented categories included class I.A (degradation of small molecules; P−value = 0.009), insertion sequences (IV.B; P−value<0.001) and conserved hypotheticals (V; P−value not significant). This pattern is also reflected in the list of highly abundant transcripts in Table 3, which also includes representatives of the ESX Type VII secretion systems [30]–[32], the extensive family of predicted proteins of unknown function sharing common N-terminal proline-glutamate (PE) and proline-proline-glutamate (PPE) motifs [10], and Type II toxin and antitoxin (TA) proteins [33].

**Table 3:** Ranking of the most abundant coding transcripts in exponential phase.

CDS	gene	class	RPKM
Rv0009	ppiA	II.A.6	878
Rv3418c	groES	III.B	814
Rv3874	esxB	VI	594
Rv0722	rpmD	II.A.1	581
Rv3460c	rpsM	II.A.1	538
Rv3477	PE31	IV.C.1	521
Rv1872c	lldD2	I.B.6a	497
Rv3408	vapC hom	V	482
Rv0685	tuf	II.A.6	479
Rv1305	atpE	I.B.8	456
Rv3462c	infA	II.A.6	452
Rv3478	PPE60	IV.C.1	441
Rv0440	groEL2	III.B	439
Rv1871c	CHP	V	372
Rv1398c	vapB10	V	352
Rv1133c	metE	I.D.2	351
Rv1158c	CHP	V	350
Rv3804c	fbpA	I.H.3	330
Rv3875	esxA	II.C.2	307
Rv3614c	espD	V	304
Rv0733	adk	I.F.5	299
Rv3407	vapB hom	V	298
Rv3852	hns	II.A.4	289
Rv1390	rpoZ	II.A.7	287
Rv3615c	espC	V	284
Rv1793	esxN	VI	271
Rv1626	two-comp	I.J.2	271
Rv3461c	rpmJ	II.A.1	247
Rv3153	nuoI	I.B.6a	246
Rv0250c	CHP	V	244
Rv3678A	CHP	I.J.1	232
Rv2840c	CHP	V	230
Rv0700	rpsJ	II.A.1	224
Rv1630	rpsA	II.A.1	220
Rv2472	CHP	V	218
Rv0705	rpsS	II.A.1	213
Rv0831c	CHP	V	210
Rv2352c	PPE38	IV.C.1	206
Rv1886c	fbpB	I.H.3	206
Rv1306	atpF	I.B.8	205
Rv1072	CHP	II.C.5	203
Rv3648c	cspA	III.E	199
Rv3312A	pilin	V	199
Rv3459c	rpsK	II.A.1	196
Rv1233c	CHP	V	194
Rv2162c	PE_PGRS38	IV.C.1	191
Rv1310	atpD	I.B.8	190
Rv2348c	HP	VI	188
Rv1638A	CHP	V	188
Rv1397c	vapC10	V	187

HP: hypothetical protein; CHP: conserved hypothetical protein; TA: toxin-antitoxin pair.

#### Stationary phase

The stationary phase samples displayed a profile very different to that observed in exponential cultures, with only 421 CDSs (11% of the genome) expressed at a level of RPKM ≥5. The coding transcriptome was dominated by genes belonging to the DosR regulon that has been extensively characterized in the context of the adaptive response of *M. tuberculosis* to a hypoxic environment [5], [8] (reviewed in [34]), with 23 of the 50 most highly-expressed genes linked to DosR (Table 4). For all genes with RPKM ≥5 in either direction, 383 were shared between the two growth phases, 2,753 genes were specifically expressed in exponential phase and 38 were specific for stationary phase.

**Table 4:** Ranking of the most abundant coding transcripts in stationary phase.

CDS	gene	class	RPKM
*Rv2031c	acr1	III.B	6566
*Rv2626c	hrp1	V	2768
*Rv3131	CHP	V	822
*Rv3130c	tgs1	V	594
*Rv2623	usp	V	409
*Rv2032	acg	V	391
*Rv1733c	CHP	II.C.5	315
Rv1398c	vapB10	V	308
*Rv2007c	fdxA	I.B.6c	263
Rv2159c	CHP	V	193
*Rv2625c	CHP	II.C.5	189
*Rv2624c	usp	V	184
*Rv3127	CHP	V	171
*Rv2030c	CHP	V	156
*Rv3134c	usp	V	154
*Rv1737c	narK2	III.A.4	151
*Rv1996	usp	V	147
Rv3841	bfrB	I.G.14	142
Rv3615c	espC	V	141
*Rv2628	HP	VI	134
*Rv1738	CHP	V	129
*Rv2627c	CHP	V	117
Rv3614c	espD	V	110
Rv2137c	CHP	V	106
Rv2161c	CHP	V	91
Rv1535	HP	VI	87
Rv3133c	dosR	I.J.2	79
Rv0824c	desA1	I.H.2	79
Rv1736c	narX	I.B.6b	77
Rv1793	esxN	VI	71
*Rv0079	HP	VI	70
*Rv2629	CHP	V	68
Rv0276	CHP	V	62
Rv3874	esxB	VI	60
*Rv0569	CHP	V	59
Rv1732c	redox	V	59
Rv1872c	lldD2	I.B.6a	56
Rv3616c	espA	V	54
Rv2780	ald	I.A.2	54
Rv1623c	cydA	I.B.6c	46
Rv3241c	CHP	II.A.1	46
Rv2158c	murE	II.C.3	45
Rv2160c	tetR	V	45
*Rv0080	CHP	V	43
Rv3408	vapC hom	V	38
*Rv0572c	HP	VI	38
Rv2160A	tetR	V	37
Rv3875	esxA	II.C.2	36
Rv3230c	redox	VI	35
Rv3371	tgs	V	34

HP: hypothetical protein; CHP: conserved hypothetical protein; TA: toxin-antitoxin pair; usp: universal stress protein family.

*DosR regulon.

### The non-coding transcriptome

#### Antisense transcripts

Antisense reads mapped widely across the genome. For the 3,136 genes with RPKM ≥5 in exponential culture, the median level of antisense transcription was 13% of that in the sense orientation, though a subset of 435 genes had an antisense to sense RPKM ratio ≥0.5, and 168 genes had a corresponding ratio ≥2 (Table S2). Amongst the genes with antisense:sense ≥0.5, the representation of functional classes was the inverse of the pattern observed for the coding transcriptome, with enrichment for conserved hypothetical and unknown proteins (classes V and VI, P−value = 0.0027 and 0) and members of the PE/PPE family (class IV.C, P−value not significant), and under-representation of genes involved in macromolecule biosynthesis and energy generation (classes II.A, I.B and II.B, P−value = 0.022, 0.022 and 0.002) (Figure 1B).

**Figure 2:**
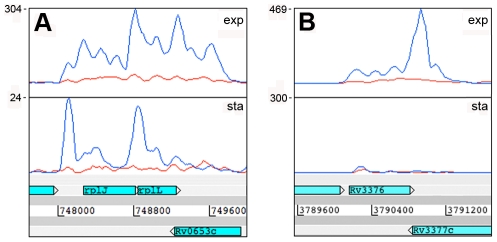
Antisense transcripts arising from 3’ UTRs. Examples of transcription profiles vizualised using the Artemis genome browser. Blue and red traces show transcription in the forward and reverse direction respectively. The x-axis records the position on the genome, and the y-axis records the number of reads mapped at that location; the maximum expression is shown on the y-axis. Examples illustrate 3’ UTR profiles that tail off into the adjacent gene (panel A) or rise to a peak in the overlap region (panel B). A. Rv0653c (a predicted transcriptional regulator) is covered by an antisense transcript extending from the 3’ end of the *rplJ*-*rplL* ribosomal protein operon in exponential phase, but not in stationary phase. B. The 3’ end of Rv3377c (a putative cyclase gene) is covered by an antisense transcript from the converging gene Rv3776. Again the antisense overlap is present only in the exponential phase.

To determine the origin of the antisense transcripts, we examined 150 CDSs that were mapped by 500 or more antisense reads, accounting for 27% of the total antisense transcriptome. For 39 of the CDSs, the antisense signal was distributed throughout the length of a highly expressed gene and represented less than 20% of the sense signal. This profile suggests non-specific antisense background that may include spurious second strand synthesis during reverse transcription. For two thirds of the remaining genes with abundant antisense, visual inspection of transcriptional profiles using the Artemis genome browser [35] indicated that antisense transcripts were associated with the 3’ UTR of an oppositely oriented adjacent or overlapping gene (Figure 2). The most abundant 3’ UTR-derived antisense transcripts are listed in Table 5. The *M. tuberculosis* genome contains 502 convergent 3’-3’ gene pairs with intergenic separation of 100 nucleotides or less. The functional class representation amongst these genes showed no significant difference from the overall genome, though it includes a disproportionate number of toxin genes (42 from a total of 88). The 3’-3’ subset is significantly enriched in antisense transcripts – including 311 of the 435 genes with antisense:sense ratio ≥0.5 (P−value<0.01) – with a significant over-representation of cell envelope-associated class II.C amongst the genes covered by antisense (Fisher's exact one side test, P−value = 0.034)(Figure S1). While 3’ UTRs make a significant contribution to the antisense transcriptome, we found only one instance in which a 5’ UTR generated a visible antisense overlap. In this case the 5’ UTR of the *infC* gene (Rv1641) overlapped with the 5’ end of the divergently oriented *lysX*.

**Figure 3:**
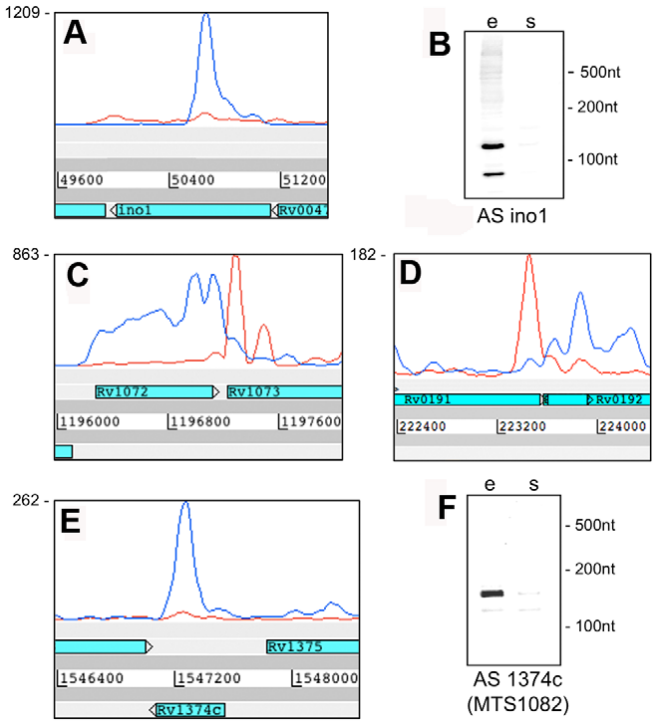
Antisense transcripts independent of 3’ UTRs. Artemis profiles are shown for antisense transcripts that cover the middle (A) or either end of a gene (C, D), or that cover the entire CDS (E). Panels B and F illustrate further characterization of the *ino1* (Rv0046, inositol-1-phosphate synthase) and Rv1374c antisense transcripts by Northern blotting.

A second category of antisense transcripts arose within genes. These ranged in length from short sequences covering the middle or either end of a CDS to longer sequences covering one or more genes (Figure 3 and Table 6). For four of the genes in Table 6, transcription was detected almost exclusively in the antisense orientation. Some of these may represent mis-annotations; Rv2081c is annotated as encoding a hypothetical protein, for example, though the antisense transcript can be matched to a potential open reading frame on the opposite strand. The antisense transcript generated from the 3’ end of Rv2816c, encoding the CRISPR (Clustered Regularly Interspaced Short Palindromic Repeats)-associated protein (*cas2*) [36], [37], extends over more than a kilobase, covering most of the adjacent Rv2817c *cas1* gene. Rv1374c is annotated as encoding a hypothetical protein, but Northern blot analysis of the antisense transcript (Figure 3F) and absence of an appropriately oriented open reading frame, suggests that this may represent an intergenic small RNA; this also applies to Rv1734c (see below). It is likely that the antisense transcript mapped to Rv1805c corresponds to an M-box riboswitch predicted in the 5’ UTR of Rv1806 [38] (discussed below).

**Table 5:** Ranking of most abundant antisense transcripts derived from 3’ UTRs.

Antisense to	gene	class	3’ UTR of	gene	class	AS RPKM	AS coverage
Rv0758	phoR	I.J.2	Rv0759c	CHP	V	153	3’ end
Rv0463	CMP	II.C.4	Rv0464c	CHP	V	152	whole
Rv0128	CMP	II.C.5	Rv0129c	fbpC	I.H.3	139	3’ end
Rv0502	acyltransferase	V	Rv0503c	cmaA2	I.H.2	135	3’ end
Rv0200	CMP	II.C.5	Rv0201c	CHP	V	111	3’ end
Rv0866	moaE2	I.G.4	Rv0867c	rpfA	VI	95	3’ end
Rv0478	deoC	I.F.4	Rv0479c	CMP	II.C.4	91	3’ end
Rv2464c	DNA glycosylase	II.A.5	Rv2463	lipP	II.B.5	91	3’ end
Rv2219A	CMP	II.C.4	Rv2219	CMP	II.C.5	88	whole
Rv2985	mutT1	II.A.5	Rv2986c	hupB	II.A.4	87	3’ end
Rv0882	MP	II.C.5	Rv0883c	CHP	V	80	whole
Rv2375	CHP	V	Rv2376c	cfp2	VII	80	whole
Rv3409c	choD	I.A.3	Rv3408	vapC hom	V	75	whole
Rv1132	CMP	II.C.5	Rv1133c	metE	I.D.2	75	3’ end
Rv0653c	tetR	I.J.1	Rv0652	rplL	II.A.1	74	whole
Rv3377c	cyclase	VI	Rv3376	hydrolase	V	73	3’ end
Rv0343	iniC	VI	Rv0344	lpqJ	II.C.1	70	3’ end
Rv1773c	iclR	I.J.1	Rv1772	HP	VI	64	3’ end
Rv0010	CMP	II.C.4	Rv0009	ppiA	II.A.2	40	3’ end

Ranking of most abundant antisense transcripts during exponential growth; MP/CMP: membrane protein/conserved membrane protein.

Functional class II.C (cell wall and cell envelope) is significantly over-represented (P−value = 0.004).

**Table 6:** Ranking of most abundant antisense transcripts independent of 3’ UTRs.

CDS	gene	class	AS RPKM	AS/sense RPKM	AS coverage
Rv2816c	cas2	V	256	34.6	whole**
Rv2081c	MP	II.C.5	240	14.1	whole
Rv0842	transporter	II.C.5	205	4.7	5’ end
Rv0046c	ino1	VII	191	1.7	middle
Rv0846c	oxidase	VII	169	0.7	middle
Rv3221A	rshA	II.A.7	106	3.7	whole
Rv1389	gmk	I.F.1	91	0.2	5’ end
Rv2082	CHP	V	82	<0.2	3’ end
Rv0460	CHP	VI	81	2.4	whole
Rv0343	iniC	VI	75	2.7	middle
Rv1179c	helicase	VI	74	0.8	middle
Rv1847	CHP	V	73	5.0	middle
Rv1073	CHP	V	68	1.3	5’ end
Rv1374c	HP	VI	58	20.7	whole
Rv0191	transporter	II.C.5	56	0.9	3’ end
Rv1805c	HP	VI	55	46.9	whole
Rv1162	narH	I.B.6b	53	0.7	middle
Rv3634c	galE1	I.C.3	52	0.8	middle
Rv0583c	lpqN	II.C.1	23	0.6	5’ end

Ranking of most abundant antisense transcripts during exponential growth.

**antisense transcript extends over multiple genes.

#### Intergenic transcripts

To determine the origin of transcripts mapping to IGRs we examined the subset of IGRs longer than 100 nucleotides that were mapped by at least 500 reads. This included 141 IGRs, accounting for 56% of the total intergenic reads in exponential phase (after removal of reads mapping to *rrn*, *ssr* and *rnpB*). In stationary phase only 24 of the IGRs displayed 500 reads or more, and half of these were shared between exponential and stationary phase (Table S3). Visual inspection of Artemis traces identified 5’ UTRs and sRNA candidates as the dominant sources of intergenic reads, with 3’ UTRs, tRNAs and spill-over from antisense transcripts making smaller contributions (Table 7). Contiguous transcriptional activity was observed across the entire IGR in some cases, precluding a clear distinction between the potential contribution of 5’ and 3’ UTRs; we have systematically assigned these as 5’ UTRs in the present analysis.

**Table 7:** Analysis of abundant intergenic transcripts.

category	no. of IGRs (%)	no. of reads (%)
5' UTR	97 (69)	127 478 (44)
3' UTR	17 (12)	12 680 (4)
sRNA	15 (11)	137 287 (48)
tRNA	8 (6)	6 973 (2)
antisense	4 (3)	3 313 (1)
total	141	287 731

IGRs ≥100 nucleotides, mapped by ≥500 reads during exponential growth.

**Figure 4:**
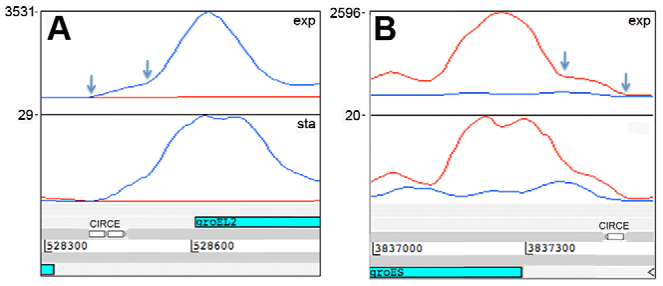
5’ UTRs of heat shock chaperones. Transcription of *groEL2* (panel A) and *groES* (panel B) is associated with long 5’-leader sequences that include the CIRCE motifs involved in heat shock regulation. Arrows indicate the putative transcription start sites based on signal start and increase in slope, respectively, suggesting that there are CIRCE-dependent and CIRCE-independent transcription start sites for these genes. Genome locations are indicated below and maximum expression in exponential and stationary phases are shown on the y-axis.

The representation of functional groups amongst the 82 genes with a prominent 5’ UTR broadly parallels that seen for the overall coding transcriptome, with a marked enrichment of class II.A genes involved in macromolecule synthesis (Figure 1C). The most abundant intergenic 5’ UTRs longer than 150 nucleotides are ranked by RPKM in Table 8, and include a high proportion of genes involved in transcription and translation. Figure 4 illustrates the 5’ UTRs associated with genes encoding the heat shock chaperones GroES and GroEL2, showing a biphasic profile in which a subset of transcripts include the CIRCE elements implicated in transcriptional regulation by HrcA [39].

**Table 8:** Ranking of most abundant 5’ UTRs in intergenic regions.

						UTR/CDS RPKM	
CDS	gene	class	CDS RPKM	UTR length**	UTR RPKM	exp	sta***
Rv3418c	groES	III.B	814	267	549	0.7	0.5
Rv0440	groEL2	III.B	439	226	542	1.2	1.0
Rv3053c	nrdH	I.F.3	169	388	376	2.2	9.1
Rv1297	rho	II.A.7	156	257	364	2.3	(0.3)
Rv3462c	infA	II.A.6	452	204	349	0.8	0.8
Rv2391	nirA	I.B.6b	146	284	303	2.1	(2.6)
Rv1641	infC	II.A.6	115	326	295	2.6	4.9
Rv3477	PE31	IV.C.1	521	156	276	0.5	0.4
Rv0667	rpoB	II.A.7	71	360	245	3.5	10.1
Rv1133c	metE	I.D.2	351	352	233	0.7	0.6
Rv3460c	rpsM	II.A.1	538	216	223	0.4	(1.0)
Rv1396c	PE_PGRS25	IV.C.1	16	255	223	14.2	4.3
Rv0597c	CHP	V	17	251	211	12.7	16.1
Rv1095	phoH2	I.A.4	44	210	208	4.7	11.9
Rv0107c	ctpI	III.A.2	19	354	194	10.2	10.1
Rv0685	tuf	II.A.6	479	231	171	0.4	0.3
Rv1535	HP	II.A.3	21	565	169	8.2	0.7
Rv3847	HP	VI	45	211	159	3.5	2.3
Rv1536	ileS	II.A.3	21	307	155	7.6	10.9
Rv3457c	rpoA	II.A.7	119	152	150	1.3	(1.7)

IGRs ≥100 nucleotides, mapped by ≥500 reads.

**length is given in nucleotides and according to visual inspection using Artemis browser.

***Values in brackets indicate that RPKMs for 5’ UTR as well as for CDS was lower than 5; exp = exponential; sta = stationary.

**Figure 5:**
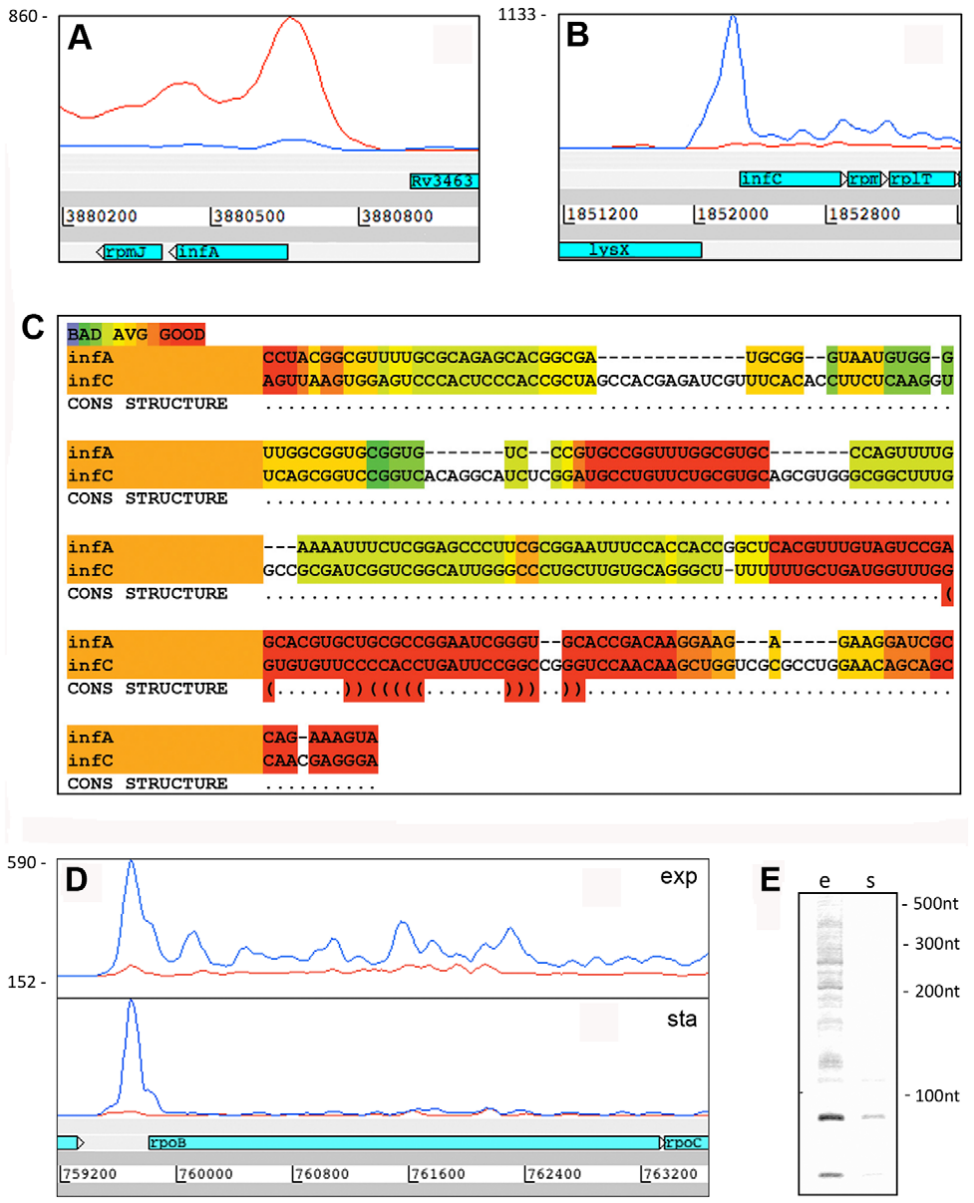
5’ UTRs from genes involved in transcription and translation. Panels A and B show Artemis profiles of the 5’ UTRs of the *infA* and *infC* genes encoding translation initiation factors. Panel C is a T-coffee consensus alignment of these two 5’ UTRs generated using the WAR webserver (http://genome.ku.dk/resources/war) [40]. The alignment is based on the consensus from four independent alignment approaches, and shows significant homology in several regions (key to the degree of alignment is shown in the top left corner of the panel). The 5’ UTR homologies are consistent with a common recognition site involved in coordinate regulation. Panels D and E illustrate analysis of the 5’ UTR of *rpoB* by Artemis profiling and Northern blotting of samples from exponential (“e”) and stationary (‘s”) growth phases. The 5’ UTR of *rpoB* remains prominent in stationary phase, in contrast to decreased expression of the CDS.

Long 5’ UTRs in bacteria are characteristically associated with regions of secondary structure that regulate mRNA stability and translation, often by interaction with RNA, proteins or small molecule ligands [13]. Alignment of the long, highly-expressed 5’ UTRs of *infA* and *infC* using the WAR webserver [40] revealed regions of extensive homology suggesting that these genes may share some post-transcriptional regulation in *M. tuberculosis* (Figure 5A+B). Similarly, alignment of 5’ UTRs of *rpoA* and *rpsM*, which are co-transcribed in *E. coli* [41], revealed extensive sequence homology, consistent with a role in coordinate regulation (Figure S2). The *rpoB* 5’ UTR remained prominent in the stationary phase transcriptome, in contrast to the much reduced signal from the coding gene (Figure 5D+E), suggesting a potential involvement in some form of transcriptional attenuation.

**Figure 6:**
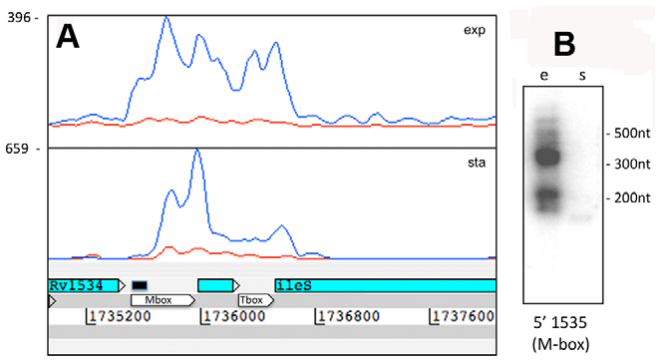
Rv1535 is flanked by two riboswitches. The region between Rv1534 and Rv1536 (*ileS,* isoleucyl-tRNA synthase) is predicted to contain two riboswitches; an Mbox upstream of Rv1535 and a T-box upstream of *ileS*. The intervening open reading frame, Rv1535, is annotated as expressing a hypothetical protein with unidentified homology or function. Panel A shows Artemis expression profiles and panel B illustrates Northern blot analysis in exponential (“e”) and growth stationary phase (“s”), using the probe indicated by a black bar above the Mbox. Riboswitch expression is evident in the form of short RNA transcripts with a more downstream start site in stationary phase.

Several riboswitches have been identified in *M. tuberculosis* by sequence homology to other organisms, and a cobalamin riboswitch has been experimentally verified (Table S4) [38], [42], [43]. Riboswitches are RNA sequences that have the ability to adopt a ligand-dependent secondary structure that has the effect of attenuating transcription or translation of downstream sequences. Riboswitch-mediated attenuation generates truncated 5’ UTR transcripts; a pattern seen for several of the genes listed in Table 8. Rv1535 and Rv1536 are predicted to harbour riboswitches in their 5’ UTRs; a metal ion-dependent M-box in the case of Rv1535 [38] and a T-box responsive to uncharged tRNAs in the case of tRNA synthase-encoding Rv1536 [42]. Analysis of the Rv1535/6 locus shows accumulation of short upstream transcripts consistent with a riboswitch in the “off” position (Figure 6). High 5’ UTR:CDS ratios suggest further candidate riboswitches amongst the genes listed in Table 8.

IGR-associated 3’ UTRs were similar to the antisense 3’ UTRs discussed above, including “tail” and “peak” profiles (illustrated in Figure 3) and sometimes extending across the IGR into the next CDS. 3’ UTRs ranged in length from 117 to 676 nucleotides with a median of 320 nucleotides. Table 9 lists the most abundant intergenic 3’ UTRs ranked by RPKM. **Intergenic sRNAs.** The Illumina technology we used involved a size fractionation step, selecting 200–250 nucleotide cDNA fragments for sequencing. To include short transcripts we included an RNA ligation step before cDNA synthesis (see Materials and Methods for details). Transcription profiles for 15 of the abundantly expressed IGRs in exponential phase were consistent with their identification as intergenic sRNAs, with short transcripts unlinked to adjacent CDSs and lacking an obvious open reading frame. These are listed in Table 10, together with selected additional examples of lower abundance sRNAs identified in other screens. We have given each of these a designation of “MTS”, for *M. tuberculosis* sRNA, with a number corresponding to a previously described IGR annotation (see Table 10 legend). We have characterized three sRNAs in more detail. Northern blot confirmation of additional examples is shown in Figure S3.

**Table 9:** Ranking of most abundant 3’ UTRs in intergenic regions in exponential phase.

				RPKM		
CDS	gene	class	UTR length**	CDS	UTR	UTR/CDS
RV1174c	TB8.4	VI	650	127	153	1.2
Rv0129*	fbpC	I.H.3	280	147	139	0.9
Rv0872c	PE_PGRS15	IV.C.1	190	60	97	1.6
Rv1919c	CHP	II.C.2	282	68	90	1.3
Rv2220	glnA1	I.D.1	427	184	69	0.4
Rv0009	ppiA	II.A.6	450	878	62	0.1
Rv1747	transporter	II.C.5	163	36	62	1.7
Rv0655	mkl	III.A	280	185	55	0.3
Rv1614	lgt	III.D	675	72	51	0.7
Rv3822	CHP	V	396	134	36	0.3
Rv3001c	ilvC	I.D.7	388	59	34	0.6
Rv3528c	HP	VI	360	23	30	1.3
Rv2520c	CMP	II.C.5	463	73	29	0.4
Rv3136	PPE51	IV.C.1	457	92	28	0.3
Rv1438	tpi	I.B.1	458	29	26	0.9
Rv1156	CHP	V	610	75	23	0.3
Rv3527	HP	VI	372	5	23	4.6

*3’ UTR extends into the adjacent CDS as an antisense transcript (Table 3A).

**length is given in nucleotides and according to visual inspection using Artemis browser.

**Table 10:** Intergenic sRNA candidates ranked according to RPKM.

sRNA*	strand	flanking CDS		RPKM			Verified§	Reference
				exp	sta			
MTS2823	F	Rv3661	Rv3662c	6748		43600	+	
MTS0900	R	Rv1144	Rv1145	312		49		
MTS2292	F	Rv2975c	Rv2976c	160		6		
MTS0997	R	Rv1264	Rv1265	155		241	+	[23]
MTS2255	F	Rv2929	Rv2930	92		4		
MTS2458	R	Rv3190c	Rv3191c	68		2		
MTS1082	F	Rv1373	Rv1375	79		4	+	
MTS1180	R	Rv1510	Rv1511	54		1		
MTS0903	R	Rv1147	Rv1148c	42		9	+	
MTS0824	F	Rv1053c	Rv1054	42		2		
MTS1827	R	Rv2355	Rv2356c	36		10		
MTS0194	F	Rv0243	Rv0244c	22		1	+	F6 [22]
MTS1635	F	Rv2108	Rv2109c	22		1		
MTS2977	R	Rv3845	Rv3846	22		0		
MTS2362	F	Rv3059	Rv3060c	21		1		
MTS1310	R	Rv1689	Rv1690	21		3	+	G2 [22]
MTS2975	F	Rv3843c	Rv3844	19		1	+	
MTS2822	R	Rv3660c	Rv3661	15		2	+	B11 [22]
MTS0858	R	Rv1092c	Rv1093	14		24	+	
MTS1338	F	Rv1733c	Rv1735c	5		116	+	
MTS0479	F	Rv0609A	Rv0610c	1		0	+	B55 [22]

*sRNAs are numbered according to the nomenclature used in the TIGR annotation of intergenic regions [http://cmr.jcvi.org/tigr-scripts/CMR/CmrHomePage.cgi] and Table S3; exp = exponential; sta = stationary.

**§:** Verified as a small transcript by Northern blot.

**Figure 7:**
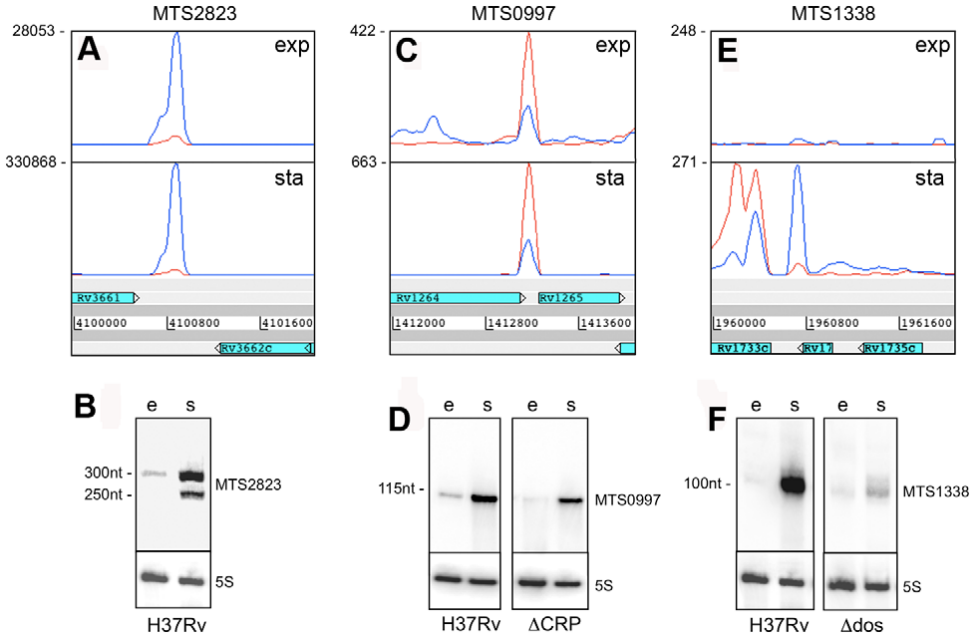
Intergenic sRNAs. Transcription profiles and Northern blots showing the expression of *M. tuberculosis* sRNAs in exponential and stationary phase.A and B. MTS2823 is the most abundant sRNA during exponential growth (“e”), with a further increase seen in stationary phase (“s”). C and D. MTS0997 is readily detectable by RNA-seq and by Northern blot during exponential growth, with a significant increase in stationary phase. Expression of MTS0997 was reduced in both growth phases in a strain of *M. tuberculosis* lacking the cAMP receptor protein (CRP). E and F. MTS1338 is barely detectable during exponential growth, but is strongly induced in stationary phase. The sRNA transcript partially overlaps Rv1734c, which is oriented in the reverse direction to MTS1338 and annotated as encoding a hypothetical protein. Deletion of the DosR two-component regulator markedly reduces, but does not entirely eliminate stationary phase expression of MTS1338.

Second only to the signals from rRNA, the sRNA candidate encoded in IGR 2823, between Rv3661 and Rv3662c, is the most abundant transcript in exponentially growing *M. tuberculosis*. MTS2823 increased a further ten-fold in stationary phase cultures, reaching a level of 50,000 RPKM, approaching that of 23S and 16S rRNA (150–200,000 RPKM). Northern blot analysis shows MTS2823 as a major 300-nucleotide transcript in exponential and stationary phase cultures, with an additional 250-nucleotide transcript appearing during stationary phase (Figure 7A+B). The 5’ end of MTS2823 was mapped to position 4,100,669 with a putative terminator 319 nucleotides further downstream consistent with the transcript size observed on the Northern blot. We were unable to match the region upstream of this 5’ end with known promoter consensus sequences. Closely related sRNAs are present in genome sequences of a subset of actinomycetes including a range of mycobacteria and rhodococci, *Nocardia farcinica*, *Gordonia bronchialis* and *Tsukamurella paurometabola*.

MTS0997 is encoded in the IGR between Rv1264 and Rv1265, in the reverse orientation to both of the flanking CDSs. It is prominent in the exponential transcriptome and further increased in the stationary phase (Figure 7C+D). Rv1265 is a hypothetical protein induced during macrophage infection [44], while Rv1264 encodes an adenylate cyclase. The 5’ end of the MTS0997 transcript was mapped by RACE, and putative −35 and −10 promoter elements were identified. Mutations in the predicted −10 hexamer eliminated expression of a β-galactosidase reporter in *Mycobacterium smegmatis* as well as in *M. tuberculosis*, seen as loss of blue colour on plates containing X-Gal (data not shown). A putative binding site for the cAMP receptor protein (CRP) was identified 107 base pairs (10 helical turns) upstream of the predicted transcription start site (Figure S4A). Expression of MTS0997 in a CRP knockout strain (provided by Roger Buxton [45]) was almost abolished in exponential phase while slightly reduced in stationary phase, suggesting some dependence on CRP regulation in both growth phases (Figure 7).

MTS1338 is transcribed in the opposite orientation to a partially overlapping open reading frame annotated as encoding Rv1734c, a conserved hypothetical protein included in the DosR regulon [5]. We were unable to detect any expression of the predicted Rv1734c CDS in either exponential or stationary phase. There was very little expression of MTS1338 in the exponential phase, but it is amongst the most abundant transcripts in the stationary phase transcriptome (Figure 7E+F). The major transcription start site was mapped to position 1,960,667, with a second minor start site further upstream at 1,960,601; three DosR binding sites are present between the main start of MTS1388 and the adjacent DosR-regulated Rv1733c [46] (Figure S4B). In a DosR knockout strain (provided by David Sherman [5]) stationary phase expression of MTS1338 was markedly reduced, though not completely eliminated (Figure 7F).

### Functional characterization of MTS2823

**Figure 8:**
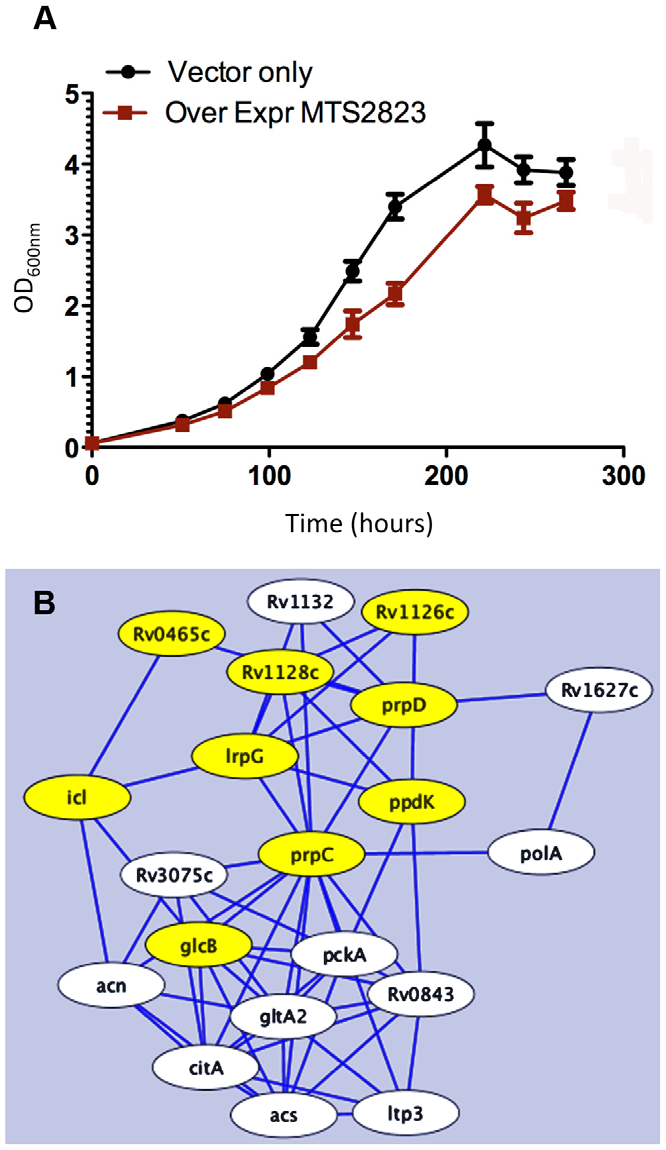
Over-expression of MTS2823. Panel A shows in vitro growth curves for *M. tuberculosis* H37Rv over-expressing MTS2823 compared to *M. tuberculosis* H37Rv harbouring the empty vector. Panel B: Hypothetical protein association network centered around *prpC*. The figure was created using the STRING database [47]. Proteins are shown as nodes and associations as lines. The methyl citrate genes *prpC*, *prpD* and their regulator, *lrpG*, are shown along with their immediate first neighbours; genes down-regulated between 2- and 2.5-fold are shown in white and genes down-regulated 2.5-fold or more are shown in yellow. Network construction and visualisation was performed in Cytoscape [77].

To explore the functional role of the dominant stationary phase sRNA, MTS2823 we engineered its over-expression in exponential phase cultures of *M. tuberculosis* H37Rv under the control of a strong rRNA promoter. Over-expression of MTS2823 had a slight but clear effect on the growth rate (Figure 8A). However, microarray analysis revealed a striking overall down-regulation of multiple genes with 301 genes showing ≥2.5-fold change (P−value ≤0.05) (Figure S6, Table S5). Apart from MTS2823 itself (represented by microarray probe MtCDC1551-3762), only two genes were up-regulated; Rv2035 encoding a potential activator of HspG (up 3.2-fold) and Rv3229c, encoding a fatty acyl desaturase DesA3 (up 3.1-fold).

The down-regulated subset showed a significant over-representation of genes involved in energy metabolism (class I.B; P−value<0.01 after FDR correction) as well as a trend towards over-representation of genes involved in macromolecular synthesis (class II.A) and insertion elements (Figure S5). Rv1131 (*prpC*/*gltA1*) encoding methyl citrate synthase, showed the greatest change of expression, with a 15-fold down-regulation. Related genes involved in the citrate synthase cycle, encoding methyl citrate dehydratase (Rv1130, *prpD*; down 7-fold) and an associated transcriptional regulator (Rv1129c, *lrpG*; down 5.6-fold), were similarly repressed. Analysis of associated partners identified in the STRING database [47] revealed down-regulation of an extended methyl citrate network (Figure 8B). Down-regulation of *prpC* in exponential phase cultures has previously been observed as a consequence of deletion of several sigma factors [48], [49]. *sigE, sigB, sigG* and *sigA* transcripts were all down-regulated in the MTS2823 over-expressing strain (Table S5). Five of the ten *vapC* toxin homologues were down-regulated ≥2.5-fold while none of their antitoxin partners were affected (Table S5). Quantitative RT-PCR (qRT-PCR) analysis of selected genes confirmed the pattern seen by microarray, over a more extended dynamic range (Table 11).

**Table 11:** Expression of selected genes upon over-expression of MTS2823.

Rv number	Gene name	up/down	microarray	qRT-PCR
MT3762*	MTS2823	up	14	144±47
Rv2035	*-*	up	3.2	2±0.8
Rv1131	prpC	down	15.2	115±50
Rv1130	prpD	down	6.8	63±26
Rv1129	lrpG	down	5.6	10±3
Rv0467	icl	down	4.7	7±0.9
Rv2710	sigB	down	3.1	5±0.5
Rv1127	ppdK	down	2.8	6±4
Rv0465	lrpI	down	2.6	9±4
Rv1837	glcB	down	2.6	4±1
Rv1132	-	down	2.4	3±0.8
Rv3153	nuoI	down	2.0	4±0.4
Rv2703	sigA	down	2.0	3±0.7
Rv0440	groEL2	down	1.5	2±0.2
Rv0667	rpoB	down	1.7	2±0.4
Rv1908	katG	down	1.3	1±0.5
Rv3418	groES	down	1.5	1±0.3

*MTS2823 is annotated on the reverse strand as MT3762 in CDC1551, and hence we have microarray data for this RNA.

Table shows fold up- or down-regulation of genes determined by microarrays and qRT-PCR; the latter is shown as average±standard deviation.

### Accumulation of sRNAs during infection

**Figure 9:**
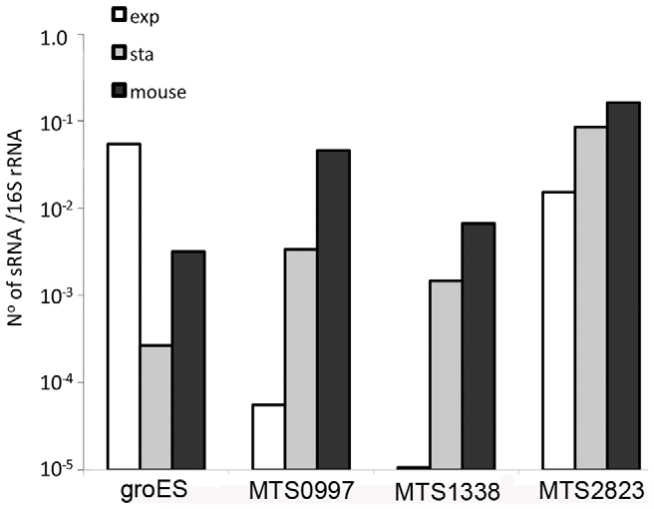
Accumulation of sRNAs during infection. Quantitative RT-PCR confirmed the findings from sequence analysis and Northern blotting that the abundance of MTS0997, MTS1338 and MTS2823 is markedly increased in stationary phase cultures in contrast to the reduction in *groES* mRNA. Analysis of *M. tuberculosis* RNA from the lungs of chronically infected mice showed a further increase in the amount of each of the sRNAs relative to 16S rRNA control.

The abundance of selected sRNAs in stationary phase cultures encouraged us to explore their expression in non-replicating *M. tuberculosis* during infection. Following aerosol infection in mice, *M. tuberculosis* undergoes a period of active replication before engagement of the adaptive immune response. Infection then persists for months with little or no change in bacterial load before mice die from cumulative lung damage. There is uncertainty as to whether the chronic phase of infection is associated with persistence of non-replicating bacteria, or a balance between bacterial replication and killing [50]–[52]. To assess expression of non-coding RNAs during the chronic stage of infection, we prepared mycobacterial RNA from lungs of mice and used qRT-PCR to measure selected sRNA and mRNA transcripts. Results were normalized to 16S rRNA.Consistent with RNA-seq and Northern blotting, qRT-PCR analysis confirmed the abundance of MTS2823 in exponential cultures with further increase in stationary phase, and the stationary phase induction of MTS0997 and MTS1338 (Figure 9). All three of the sRNAs were present at very high levels in chronically infected lung tissue; each accumulating to a level relative to 16S rRNA that was increased over and above that observed in stationary phase (Figure 9). By comparison, *groES*, a highly abundant mRNA in exponential cultures was markedly reduced in stationary phase, with an intermediate level in infected tissues suggesting the presence of replicating as well as non-replicating populations in line with the model suggested by Chao and Rubin [53] (Figure 9).

## Discussion

Application of deep sequencing technologies to study the transcriptome of *M. tuberculosis* has uncovered an abundance of non-coding RNAs including *cis*-encoded regulatory elements, antisense transcripts and intergenic sRNAs. After removal of signals from ribosomal RNAs, the percentage of reads mapping in antisense orientation and to IGRs represents 28% of the total transcriptome in exponential phase *M. tuberculosis*. This is broadly similar to the 25% reported for *Salmonella typhi* and the 27% reported for *Helicobacter pylori* [24], [25], and contributes to a growing appreciation of the prominence of non-coding RNA in bacteria. Non-coding RNAs can regulate gene expression at a post-transcriptional level, providing a level of control intermediate between conventional transcriptional control and post-translational protein turnover, that is particularly useful in the rapid response to stress stimuli and may play an important role in generation of population heterogeneity [54]. Characterisation of non-coding RNA is likely to be important in understanding the complex biology underlying tuberculosis infection [2].

Sequence-based transcriptional profiling has advantages over hybridization-based microarray analysis in displaying a greater dynamic range with single-nucleotide resolution. The number of reads mapping to individual sequences provides a realistic assessment of relative transcript abundance, although the potential for variability in the efficiency of reverse transcription precludes precise quantitation. *M. tuberculosis* has only a single ribosomal RNA operon, but rRNA represents more than 80% of total RNA content, and we compared strategies to reduce this signal by physical removal of rRNA prior to sequencing or by computational removal of rRNA prior to sequencing or computational removal of rRNA reads after sequencing. The latter approach has the advantage of limiting potential RNA degradation or otherwise skewing of the RNA composition during processing [55]. This method still provided an average level of coverage of approximately one read per nucleotide (outside *rrn*) in exponential phase samples, and was used to generate the datasets for the present analysis. To enhance representation of shorter transcripts, we included an RNA ligation step. In spite of this, RPKM values for 5S rRNA (115 nucleotides) were between 5- and 10-fold lower than those for 16S and 23S rRNA (1,537 and 3,138 nucleotides, respectively), and only a low number of reads mapped to tRNAs (<100 nucleotides), Therefore it is likely that sRNAs are under-represented, quantitatively and perhaps qualitatively, in our final transcriptome. Our classification of RNA as “non-coding” is contingent on current genome annotations, and we cannot exclude the possibility that some of these newly identified transcripts encode short peptides.

The coding transcriptome of *M. tuberculosis* reveals a profile consistent with expectations for exponentially growing bacteria, with genes involved in energy production and macromolecule biosynthesis significantly over-represented in the transcriptome as compared to the overall genome. *M. tuberculosis*-specific features include abundant representation of transcripts encoding cargo proteins exported by Type VII secretion systems [30] with genes encoding ESX-1-exported proteins ESAT6, CFP10, EspC and EspD amongst the top fifty transcripts. Transcript abundance is a feature of many, though not all of the ESX export proteins and, while bearing in mind potential changes in transcriptional regulation during infection, this may contribute to their differential immunogenicity [56]. Transcripts encoding several TA pairs are also highly abundant. The genome of *M. tuberculosis* is remarkably rich in Type II TA systems believed to modulate gene expression through ribonuclease activity [33], but their role in mycobacterial physiology and pathogenesis remains unclear. The stationary phase transcriptome is dominated by an abundance of genes induced as part of the DosR regulon that has been extensively characterized in response to a hypoxic environment [5], [8]. We had not set out to induce hypoxic conditions and we were surprised by the strength of the DosR signal. Enhanced regular aeration of cultures resulted in increased bacterial growth in our culture system, however, and we assume that the stationary phase arrest seen in this study reflects some level of oxygen depletion.

The distribution of abundant transcripts across the antisense transcriptome is largely the inverse of the coding transcriptome, with energy metabolism and macromolecule synthesis under-represented at the expense of conserved hypotheticals and proteins of unknown function. Reflecting a pattern that is emerging from a range of sequence-based bacterial transcriptome studies [57] the antisense transcriptome of *M. tuberculosis* includes a diverse size-range of transcripts arising both within genes and as a result of 3’ UTR overlaps between convergent gene pairs.

The abundance of long 3’ UTRs observed in *M. tuberculosis* may be due to inefficient termination resulting from replacement of the characteristic bacterial L-shaped intrinsic transcriptional terminator consisting of a hairpin loop followed by a poly-U stretch [58] by an I-shaped terminator lacking a poly-U stretch [59], [60]. 3’ UTR antisense transcripts have the potential to provide a regulatory connection between neighbouring genes [57] and enrichment of cell envelope and toxin genes in 3’-3’ convergent pairs may reflect an organizational motif that has functional consequences for gene expression. Several antisense transcripts are associated with foreign genetic elements, including an abundant transcript that maps to CRISPR-association genes. The CRISPR locus is a non-coding RNA defence system that bacteria use to combat invasion by foreign genetic sequences [36], [37]. In *E. coli* the CRISPR-associated (*cas*) proteins involved in this process are silenced by the H-NS DNA-binding protein during exponential growth [61] and the antisense transcript extending across *cas*-encoding Rv2816c and Rv2817c may perform an analogous regulatory function in *M. tuberculosis*.

Although we had not specifically enriched our libraries for primary 5’ ends [25] we were able to estimate the minimum length of most 5’ UTRs by examining Artemis profiles, and identify multiple very long (≥150 nt) 5’ UTRs. By analogy with other bacteria, we anticipate that these will play a role in coordination of gene expression and in regulation by small molecule ligands. Many of the genes with long, highly expressed 5’ UTRs encode ribosomal proteins or other factors involved in translation. In *Escherichia coli* coordinated expression of ribosomal RNA, ribosomal proteins and ribosome-associated factors involves the interaction of ribosomal proteins with the 5’ UTR of mRNAs causing attenuation of expression [41]. Similarly, in *B. subtilis* 5’ UTR binding by ribosomal protein L20 controls the expression of the *infC* operon [62] In *M. tuberculosis* sequence alignments of 5’ UTRs of *rpoA* with *rpsM* and of *infA* with *infC* suggest that the upstream sequences may represent elements involved in coordinate regulation of core transcriptional and translation processes, analogous to the systems described in *E. coli* and *B. subtilis* [41], [62]. The 5’ UTRs of *groE* genes overlap the CIRCE elements that are recognized by the HrcA transcriptional repressor [39] but also play a role in post-transcriptional control of mRNA stability in *B. subtilis* [63] and in *Rhodobacter capsulatus* [64]. It is likely that the transcriptional control of the heat shock response in *M. tuberculosis* is complemented by an additional layer of post-transcriptional regulation via this element. A further subset of 5’ UTRs correspond to predicted riboswitches that regulate expression of their cognate gene in response to the presence or absence of small molecules. In the absence of activation signal, riboswitch-mediated attenuation generates truncated transcripts mapping to the 5’ end of genes; a profile that is associated with many of abundant 5’ UTRs identified in the non-coding transcriptome of *M. tuberculosis*. It has been suggested that 2% of *B. subtilis* genes may be regulated by riboswitches [13] and this form of regulation may have similar importance for *M. tuberculosis*. Small regulatory RNAs, generally encoded in IGRs, have provided a focus for recent interest with respect to their role in bacterial responses to environmental change and pathogenesis (reviewed in [12]). *Trans*-encoded sRNAs typically function by base-pairing with a panel of mRNA targets in a manner that prevents translation and accelerates their degradation. In the case of the best-characterised examples, the interaction of sRNA with mRNA is mediated by the Hfq chaperone. *M. tuberculosis* belongs to the subset of bacteria which lack an identified Hfq homologue, but RNA-seq analysis is consistent with previous descriptions of the presence of multiple intergenic sRNAs.

To explore the functional role of sRNAs in *M. tuberculosis*, we manipulated the expression of MTS2823. This sRNA is present in all growth phases, but is expressed at very high levels during stationary phase; we reasoned that we might be able to recapitulate its stationary phase function by over-expression in exponential phase. Over-expression of MTS2823 caused widespread down-regulation of gene expression, with the most profound effect on the gene classes preferentially associated with exponential growth. Genes linked to the methyl citrate network, in particular *prpC,* and to a lesser extent *prpD* were most strongly down-regulated. This could represent a preferential targeting by MTS2823, either to reduce the utilization of substrates (i.e. propionyl-CoA and/or oxaloacetate) or to reduce the accumulation of potentially toxic intermediates such as methyl citrate. The participation of these genes within a feed-forward regulatory loop provides an alternative explanation for their amplified response [65], [66]. Methyl citrate genes are also down-regulated in response to hypoxia and DNA damage [8], [67]. During transition to stationary phase, the down-regulation of genes required for active bacterial replication is commonly mediated by expression of a 6S RNA molecule that interferes with transcription by RNA polymerase associated with the principal sigma factor [68]. An *M. smegmatis* homologue of MTS2823 was recently identified in a bioinformatics screen based on suboptimal structural criteria for 6S RNA, but detailed analysis, including lack of binding to RNA polymerase, led the authors to conclude that it is not a genuine 6S RNA homologue [69]. Our results suggest that MTS2823 may have functional homology with 6S RNA in its ability to mediate down-regulation of exponential phase genes, although the molecular mechanism remains to be clarified. *In vitro* models of mycobacterial persistence typically involve growth arrest, associated with down-regulation of a broad panel of genes and up-regulation of a more limited set of condition-specific genes [8], [70]. MTS2823 may be an important mediator of the common down-regulatory component.

We anticipate that the majority of *M. tuberculosis* sRNAs will function in post-transcriptional regulation of more restricted sets of mRNAs involved in adaptive responses to specific environmental stimuli. MTS1338 provides a characteristic example. Expression of MTS1338 is strongly induced under the conditions used to generate stationary phase cultures, by a mechanism that is at least partly dependent on the hypoxia-responsive DosRS two-component transcriptional regulator.

sRNAs involved in Hfq-mediated interactions are generally degraded along with their mRNA target [13]. In contrast, we observed accumulation rather than degradation of *M. tuberculosis* sRNAs in the stationary phase induction model. Even higher levels of sRNA accumulation were observed in the lungs of mice during chronic tuberculosis infection, with MTS2823 present at 16% of the level of 16S rRNA, MTS0997 at 5% and MTS1338 at 0.5%. The abundance of sRNAs in infected tissues suggests that they may provide useful biomarkers, and potentially important functional mediators, during the course of disease.

In summary, sequence-based analysis of the transcriptome of *M. tuberculosis* uncovers a wide range of novel non-coding RNAs with the potential to influence patterns of gene expression *in vitro* and during infection. Targeted analysis by deletion and over-expression may provide insights into the molecular pathogenesis of this important human disease.

## Materials and Methods

### Ethics statement

The NIAID DIR Animal Care and Use Program adheres to the U. S. Government Principles for the Utilization and Care of Vertebrate Animals Used in Testing, Research, and Training, the PHS Policy on Humane Care and Use of Laboratory Animals, the Guide for the Care and Use of Laboratory Animals, and the U.S. Animal Welfare Regulations. We operate in accord with NIH Policy Manual 3040-2 (Animal Care and Use in the Intramural Program) and in compliance with the provisions of the NIH's intramural Institutional Assurance (NIH IRP, PHS Assurance A4149-01) on file with the NIH Office of Laboratory Animal Welfare (OLAW). The NIH Intramural Research Program is fully accredited by the Association for the Assessment and Accreditation of Laboratory Animal Care, International.The Animal Care and Use Committee (ACUC) of the National Institute of Allergy and Infectious Diseases, Division of Intramural Research, with permit number NIH IRP, PHS Assurance A4149-01, approved the animal study protocol LCID-3E under which all animal experiments were performed.

### Oligonucleotides

Table S6 lists all oligos used in this study.

### Strains and culture conditions

*M. tuberculosis* H37Rv and *M. tuberculosis* H37Rv:: δ*dosR::kan* [5] were grown in Middlebrook 7H9 supplemented with 0.2% glycerol and 10% ADC in roller bottle culture. Exponential phase cultures were harvested at an OD_600_ 0.6 to 0.8; stationary phase cultures were harvested one week after OD_600_ had reached 1.0. *M. tuberculosis* H37Rv:: δRv3676 [45] had a significantly slower growth rate and was grown two weeks after OD_600_ 1.0 to generate stationary phase cultures. To monitor β-galactosidease activity, *M. tuberculosis* and *M. smegmatis* were grown on solid 7H9 supplemented with 25 µg/ml kanamycin and 50 µg/ml X-gal.

### Infections

Eight-week-old C57Bl/6 mice were infected with 100 colony forming units of *M. tuberculosis* H37Rv by aerosol using a BioAerosol nebulizing generator (CH Technologies Inc., Westwood, NJ) for 10 minutes and the infection was allowed to develop for 9 months by which time the CFUs had reached 7.5±2.5×10^5^. Mice were sacrificed, each lung homogenized in 5 ml Trizol reagent (Invitrogen Corporation, Carlsbad, CA) and bacteria were separated from the lysed lung tissue by centrifugation at 3500 rpm for 10 minutes at 4°C. The cell pellet was washed once in Trizol, followed by resuspension in 1 ml Trizol containing 5 µg/ml glycogen and stored at −80°C until bacterial RNA extraction.

### Cloning

*lacZ* reporter fusions were made by PCR amplification of the MTS0997 promoter region with primers P0997.f+r. The resulting fragment was inserted into pEJ414 [71]. Mutation of the −10 box was done with site-directed mutagenesis using primers SDM0997(−10).f+r and Pfu Ultra (Stratagene) according to manufacturer's instructions. Reporter plasmids were electroporated into *M. smegmatis* mc^2^155 [72] as well as *M. tuberculosis* H37Rv. Over-expression construct of MTS2823. The over-expression plasmid was made by replacing the *rrnB* promoter fragment in a previously described over-expression plasmid [22] with the region spanning −80 to −8 from the same promoter to generate pKA303. The −10 box is immediately followed by a *Hin*dIII site where the sRNAs can be inserted, which means that the sRNAs can be expressed from their own transcription start site. A 316 bp *Hin*dIII fragment containing MTS2823 (+1 to +304) was PCR amplified using primers ox2823.f and ox2823.r. The fragment was cloned into pCR-Blunt II-TOPO (Invitrogen), sequenced and subsequently sub-cloned into pKA303. The resulting plasmid was electroporated into *M. tuberculosis* H37Rv.

### RNA isolation and handling

Cultures were cooled rapidly by the addition of ice directly to the culture before centrifugation. RNA was isolated by means of the FastRNA Pro blue kit from QBiogene/MP Bio according to manufacturer's instructions. RNA was treated Turbo DNase (Ambion) until DNA free. The quality of RNA was assessed using a Nanodrop (ND-1000, Labtech) and Agilent bioanalyser. In order to enrich for small transcripts in the RNA-seq reactions, total RNA was treated with tobacco acid pyrophosphatase (TAP, Epicentre technologies) and subsequently ligated with T4 RNA ligase (Ambion) before reverse transcription. Bacteria from mouse lungs were disrupted by bead beating in Trizol in a Fastprep instrument, chloroform extracted and ethanol precipitated. Due to the low bacterial count RNA from three mice was pooled. The RNA was subsequently purified with Turbo DNase and phenol extraction before reverse transcription. Northern blots, RT-PCR for investigating co-transcription and 5’ RACE were carried out as described previously [22].

### RNA-seq

Construction of cDNA libraries and sequencing was carried out essentially as described previously [73]. Sequence reads were aligned to the reference sequence of *M. tuberculosis* H37Rv (EMBL accession code AL123456) as paired end data using BWA [74]. The orientation of the second read in correctly mapped pairs was reversed using Samtools [74] before producing coverage plots in order to maintain the directional fidelity of the data. Novel intergenic features were annotated by visual inspection of the transcriptome using the Artemis genome browser. RPKM values were calculated using only sequence reads that mapped to annotated features unambiguously (the ‘XA’ note in the alignment file was used to identify alternative mapping locations) and on the correct strand. To ascertain the proportion of a gene to which reads could be unambiguously mapped, 54 nucleotide paired end data were simulated from the genome sequence and aligned to the sequence under the settings used for mapping the RNA-seq reads.

### qRT-PCR

cDNA for quantitative RT-PCR and RNA-seq was made with random primers and Superscript III according to manufacturer's instructions (Invitrogen) with an additional incubation step for one hour at 55°C in order to optimize read-through of highly structured sequences. qRT-PCR was carried out on a 7500 Fast Real-Time PCR System (Applied Biosystems) using Fast SYBR Green Master Mix (Applied Biosystems). RNA without RT (RT-) was analyzed alongside cDNA (RT+). Standard curves were performed for each gene analyzed and the quantities of cDNA within the samples were calculated from cycle threshold values. Data were averaged, adjusted for chromosomal DNA contamination (RT+ minus RT-) and normalized to corresponding 16S RNA values.

### Microarray analysis

Microarrays were performed on three biological replicates of over-expression strain against the control strain containing empty vector. Reverse transcription, hybridization and subsequent analysis were carried out as described [75].

### Statistical analysis

All analyses where carried out using STATA s.e.m version 10. To compare the frequencies of different functional categories we used Fisher's exact test. For the comparison between the annotated genome and the transcriptome, and for the comparison of the transcriptome with the antisense transcripts we used all the categories and therefore the False Discovery Rate correction for multiple testing [76] was applied. For the rest of the analyses only selected categories were tested and no test for multiple correction was required.

## Supporting Information

Figure S1 **Representation of functional classes amongst genes involved in 3’ UTR antisense overlaps.** The difference in frequency of selected functional classes when comparing genes organized in 3’-3’ convergent pairs and having antisense to sense ratio≥0.5 (N = 285) with the total set of CDSs with RPKM≥5 (N = 3,136). Functional class II.C is over-represented (89/245) amongst genes covered by antisense transcripts (Fisher's exact one side test, P−value = 0.034).
(TIF)

Figure S2 **Alignment of long 5’ UTRs from** ***rpoA*** **and** ***rpsM.*** T-coffee consensus alignment of the 5’ UTRs of *rpoA* and *rpsM* (http://genome.ku.dk/resources/war) [40] highlights regions of homology that may play a role in the coordinated regulation of expression.
(TIF)

Figure S3 **Northern blot confirmation of additional 5’ UTRs and intergenic sRNAs.** Each panel shows Northern blots with RNA from exponential (“e”) and stationary (“s”) phase. The stationary phase profile of the 5’ UTR of Rv2950 suggests attenuation. The 5’ UTR of *dnaA* is not expressed in stationary phase. MTS0858 is prominent in both growth phases, while expression of MTS0930 and MTS2975 is reduced in stationary phase.
(TIF)

Figure S4 **Mapping of sRNA transcriptional start sites and predicted transcription factor binding sites.** Panel A shows the region surrounding MTS0977. The 5’ end (not TAP specific) was mapped to the G nucleotide shown by an asterisk. Putative promoter elements as well as CRP binding site (CBS) are outlined by rectangles. The 3’ end is approximate, based on the size of the transcript (as judged by Northern blotting) and structural predictions (using Mfold). The start of Rv1265 was mapped to the −35 element of MTS0997.Panel B shows RLM-RACE result for mapping the transcription start sites of MTS1338 (using primer 1338.R, and indicated by asterisks above the sequence) and the region surrounding MTS1338 with DosR binding sites according to [46] indicated.
(TIF)

Figure S5 **Distribution of down-regulated genes upon over-expression of MTS2823.** Genes involved in energy metabolism (class I.B) are over-represented among down-regulated genes. Genes identified as down-regulated ≥2.5-fold by microarray analysis were grouped according to the functional class of their predicted gene product as assigned in the original genome annotation [10]. Values on the x-axis represents a difference in percentage, positive values indicate over-representation of a particular functional class whereas negative values indicate under-representation. Asterisks indicate P−value<0.01 after FDR correction.
(TIF)

Figure S6 **Effect of MTS2823 over-expression on** ***M. tuberculosis*** **genes.** The figure shows a Volcano plot of the microarray data, which illustrates how the majority of genes are down-regulated as a result of MTS2823 over-expression.
(TIF)

Table S1 **Pairwise correlation coefficients between five exponential phase samples based on sense and antisense RPKM.**
(DOC)

Table S2 **Sense and antisense reads and RPKM scores for all CDSs (supplied as Excel file).**
(XLS)

Table S3 **Reads mapped to intergenic regions**> **50 nucleotides (supplied as Excel file).**
(XLS)

Table S4 **Expression pattern of predicted riboswitches.**
(DOC)

Table S5 **Expression data for genes with fold-change**≥ **2.5 upon over-expression of MTS2823 (supplied as Excel file).**
(XLS)

Table S6 **Oligonucleotides used in the study.**
(XLS)

## References

[1] WHO (2009). Global tuberculosis control - epidemiology, strategy, financing..

[2] Barry CE, 3rd, Boshoff HI, Dartois V, Dick T, Ehrt S (2009). The spectrum of latent tuberculosis: rethinking the biology and intervention strategies.. Nat Rev Microbiol.

[3] Reddy TB, Riley R, Wymore F, Montgomery P, DeCaprio D (2009). TB database: an integrated platform for tuberculosis research.. Nucleic Acids Res.

[4] Manganelli R, Voskuil MI, Schoolnik GK, Dubnau E, Gomez M, et al. (2002). Role of the extracytoplasmic-function sigma factor sigma(H) in *Mycobacterium tuberculosis* global gene expression.. Mol Microbiol.

[5] Park HD, Guinn KM, Harrell MI, Liao R, Voskuil MI (2003). Rv3133c/dosR is a transcription factor that mediates the hypoxic response of *Mycobacterium tuberculosis*.. Mol Microbiol.

[6] Rustad TR, Harrell MI, Liao R, Sherman DR (2008). The enduring hypoxic response of Mycobacterium tuberculosis.. PLoS One.

[7] Schnappinger D, Ehrt S, Voskuil MI, Liu Y, Mangan JA (2003). Transcriptional Adaptation of *Mycobacterium tuberculosis* within Macrophages: Insights into the Phagosomal Environment.. J Exp Med.

[8] Voskuil MI, Schnappinger D, Visconti KC, Harrell MI, Dolganov GM (2003). Inhibition of respiration by nitric oxide induces a *Mycobacterium tuberculosis* dormancy program.. J Exp Med.

[9] Walters SB, Dubnau E, Kolesnikova I, Laval F, Daffe M (2006). The *Mycobacterium tuberculosis* PhoPR two-component system regulates genes essential for virulence and complex lipid biosynthesis.. Mol Microbiol.

[10] Cole ST, Brosch R, Parkhill J, Garnier T, Churcher C (1998). Deciphering the biology of *Mycobacterium tuberculosis* from the complete genome sequence.. Nature.

[11] Gripenland J, Netterling S, Loh E, Tiensuu T, Toledo-Arana A (2010). RNAs: regulators of bacterial virulence.. Nat Rev Microbiol.

[12] Papenfort K, Vogel J (2010). Regulatory RNA in bacterial pathogens.. Cell Host Microbe.

[13] Waters LS, Storz G (2009). Regulatory RNAs in bacteria.. Cell.

[14] Mandal M, Breaker RR (2004). Gene regulation by riboswitches.. Nat Rev Mol Cell Biol.

[15] Loh E, Dussurget O, Gripenland J, Vaitkevicius K, Tiensuu T (2009). A trans-acting riboswitch controls expression of the virulence regulator PrfA in *Listeria monocytogenes*.. Cell.

[16] Gottesman S, Storz G (2010). Bacterial Small RNA Regulators: Versatile Roles and Rapidly Evolving Variations.. http://dx.doi.org/10.1101/cshperspect.a003798.

[17] Toledo-Arana A, Dussurget O, Nikitas G, Sesto N, Guet-Revillet H (2009). The Listeria transcriptional landscape from saprophytism to virulence.. Nature.

[18] Pfeiffer V, Sittka A, Tomer R, Tedin K, Brinkmann V (2007). A small non-coding RNA of the invasion gene island (SPI-1) represses outer membrane protein synthesis from the Salmonella core genome.. Mol Microbiol.

[19] Pichon C, Felden B (2005). Small RNA genes expressed from *Staphylococcus aureus* genomic and pathogenicity islands with specific expression among pathogenic strains.. Proc Natl Acad Sci U S A.

[20] Mandin P, Repoila F, Vergassola M, Geissmann T, Cossart P (2007). Identification of new noncoding RNAs in *Listeria monocytogenes* and prediction of mRNA targets.. Nucleic Acids Res.

[21] Arnvig KB, Young DB (2010). Regulation of pathogen metabolism by small RNA.. Drug Discov Today Dis Mech.

[22] Arnvig KB, Young DB (2009). Identification of small RNAs in *Mycobacterium tuberculosis*.. Mol Microbiol.

[23] Dichiara JM, Contreras-Martinez LM, Livny J, Smith D, McDonough KA (2010). Multiple small RNAs identified in *Mycobacterium bovis* BCG are also expressed in Mycobacterium tuberculosis and *Mycobacterium smegmatis*.. Nucleic Acids Res.

[24] Perkins TT, Kingsley RA, Fookes MC, Gardner PP, James KD (2009). A strand-specific RNA-Seq analysis of the transcriptome of the typhoid bacillus *Salmonella typhi*.. PLoS Genet.

[25] Sharma CM, Hoffmann S, Darfeuille F, Reignier J, Findeiss S (2010). The primary transcriptome of the major human pathogen Helicobacter pylori.. Nature.

[26] Sittka A, Lucchini S, Papenfort K, Sharma CM, Rolle K (2008). Deep sequencing analysis of small noncoding RNA and mRNA targets of the global post-transcriptional regulator, Hfq.. PLoS Genet.

[27] Albrecht M, Sharma CM, Reinhardt R, Vogel J, Rudel T (2010). Deep sequencing-based discovery of the *Chlamydia trachomatis* transcriptome.. Nucleic Acids Res.

[28] Bohn C, Rigoulay C, Chabelskaya S, Sharma CM, Marchais A (2010). Experimental discovery of small RNAs in *Staphylococcus aureus* reveals a riboregulator of central metabolism.. Nucleic Acids Res.

[29] Guell M, van Noort V, Yus E, Chen WH, Leigh-Bell J (2009). Transcriptome complexity in a genome-reduced bacterium.. Science.

[30] Bitter W, Houben EN, Luirink J, Appelmelk BJ (2009). Type VII secretion in mycobacteria: classification in line with cell envelope structure.. Trends Microbiol.

[31] Pym AS, Brodin P, Brosch R, Huerre M, Cole ST (2002). Loss of RD1 contributed to the attenuation of the live tuberculosis vaccines *Mycobacterium bovis* BCG and *Mycobacterium microti*.. Mol Microbiol.

[32] Skjot RL, Oettinger T, Rosenkrands I, Ravn P, Brock I (2000). Comparative evaluation of low-molecular-mass proteins from *Mycobacterium tuberculosis* identifies members of the ESAT-6 family as immunodominant T-cell antigens.. Infect Immun.

[33] Ramage HR, Connolly LE, Cox JS (2009). Comprehensive functional analysis of *Mycobacterium tuberculosis* toxin-antitoxin systems: implications for pathogenesis, stress responses, and evolution.. PLoS Genet.

[34] Rustad TR, Sherrid AM, Minch KJ, Sherman DR (2009). Hypoxia: a window into *Mycobacterium tuberculosis* latency.. Cell Microbiol.

[35] Rutherford K, Parkhill J, Crook J, Horsnell T, Rice P (2000). Artemis: sequence visualization and annotation.. Bioinformatics.

[36] Sorek R, Kunin V, Hugenholtz P (2008). CRISPR--a widespread system that provides acquired resistance against phages in bacteria and archaea.. Nat Rev Microbiol.

[37] Karginov FV, Hannon GJ (2010). The CRISPR system: small RNA-guided defense in bacteria and archaea.. Mol Cell.

[38] Gardner PP, Daub J, Tate JG, Nawrocki EP, Kolbe DL (2009). Rfam: updates to the RNA families database.. Nucleic Acids Res.

[39] Stewart GR, Wernisch L, Stabler R, Mangan JA, Hinds J (2002). Dissection of the heat-shock response in *Mycobacterium tuberculosis* using mutants and microarrays.. Microbiology.

[40] Torarinsson E, Lindgreen S (2008). WAR: Webserver for aligning structural RNAs.. Nucleic Acids Res.

[41] Lindahl L, Zengel JM (1986). Ribosomal genes in *Escherichia coli*.. Annu Rev Genet.

[42] Vitreschak AG, Mironov AA, Lyubetsky VA, Gelfand MS (2008). Comparative genomic analysis of T-box regulatory systems in bacteria.. Rna.

[43] Warner DF, Savvi S, Mizrahi V, Dawes SS (2007). A riboswitch regulates expression of the coenzyme B12-independent methionine synthase in *Mycobacterium tuberculosis*: implications for differential methionine synthase function in strains H37Rv and CDC1551.. J Bacteriol.

[44] Hobson RJ, McBride AJ, Kempsell KE, Dale JW (2002). Use of an arrayed promoter-probe library for the identification of macrophage-regulated genes in *Mycobacterium tuberculosis*.. Microbiology.

[45] Rickman L, Scott C, Hunt DM, Hutchinson T, Menendez MC (2005). A member of the cAMP receptor protein family of transcription regulators in *Mycobacterium tuberculosis* is required for virulence in mice and controls transcription of the rpfA gene coding for a resuscitation promoting factor.. Mol Microbiol.

[46] Lun DS, Sherrid A, Weiner B, Sherman DR, Galagan JE (2009). A blind deconvolution approach to high-resolution mapping of transcription factor binding sites from ChIP-seq data.. Genome Biol.

[47] Raman K, Chandra N (2008). *Mycobacterium tuberculosis* interactome analysis unravels potential pathways to drug resistance.. BMC Microbiol.

[48] Manganelli R, Voskuil MI, Schoolnik GK, Smith I (2001). The Mycobacterium tuberculosis ECF sigma factor sigmaE: role in global gene expression and survival in macrophages.. Mol Microbiol.

[49] Sun R, Converse PJ, Ko C, Tyagi S, Morrison NE (2004). *Mycobacterium tuberculosis* ECF sigma factor sigC is required for lethality in mice and for the conditional expression of a defined gene set.. Mol Microbiol.

[50] Munoz-Elias EJ, Timm J, Botha T, Chan WT, Gomez JE (2005). Replication dynamics of *Mycobacterium tuberculosis* in chronically infected mice.. Infect Immun.

[51] Gill WP, Harik NS, Whiddon MR, Liao RP, Mittler JE (2009). A replication clock for *Mycobacterium tuberculosis*.. Nat Med.

[52] Rees RJ, Hart PD (1961). Analysis of the host-parasite equilibrium in chronic murine tuberculosis by total and viable bacillary counts.. Br J Exp Pathol.

[53] Chao MC, Rubin EJ (2010). Letting sleeping dos lie: does dormancy play a role in tuberculosis?. Annu Rev Microbiol.

[54] Komorowski M, Miekisz J, Kierzek AM (2009). Translational repression contributes greater noise to gene expression than transcriptional repression.. Biophys J.

[55] Vliet v (2010). Next generation sequencing of microbial transcriptomes: challenges and opportunities.. FEMS Microiol Lett.

[56] Jones GJ, Gordon SV, Hewinson RG, Vordermeier HM (2010). Screening of predicted secreted antigens from *Mycobacterium bovis* reveals the immunodominance of the ESAT-6 protein family.. Infect Immun.

[57] Georg J, Hess WR (2011). cis-antisense RNA, another level of gene regulation in bacteria.. Microbiol Mol Biol Rev.

[58] Epshtein V, Cardinale CJ, Ruckenstein AE, Borukhov S, Nudler E (2007). An allosteric path to transcription termination.. Mol Cell.

[59] Unniraman S, Prakash R, Nagaraja V (2002). Conserved economics of transcription termination in eubacteria.. Nucleic Acids Res.

[60] Mitra A, Angamuthu K, Nagaraja V (2008). Genome-wide analysis of the intrinsic terminators of transcription across the genus *Mycobacterium*.. Tuberculosis.

[61] Pul U, Wurm R, Arslan Z, Geissen R, Hofmann N (2010). Identification and characterization of *E. coli* CRISPR-cas promoters and their silencing by H-NS.. Mol Microbiol.

[62] Choonee N, Even S, Zig L, Putzer H (2007). Ribosomal protein L20 controls expression of the *Bacillus subtilis* infC operon via a transcription attenuation mechanism.. Nucleic Acids Res.

[63] Homuth G, Mogk A, Schumann W (1999). Post-transcriptional regulation of the *Bacillus subtilis* dnaK operon.. Mol Microbiol.

[64] Jager S, Jager A, Klug G (2004). CIRCE is not involved in heat-dependent transcription of groESL but in stabilization of the mRNA 5’-end in *Rhodobacter capsulatus*.. Nucleic Acids Res.

[65] Datta P, Shi L, Bibi N, Balazsi G, Gennaro ML (2011). Regulation of central metabolism genes of *Mycobacterium tuberculosis* by parallel feed-forward loops controlled by sigma factor E (σ(E)).. J Bacteriol.

[66] Micklinghoff JC, Breitinger KJ, Schmidt M, Geffers R, Eikmanns BJ (2009). Role of the transcriptional regulator RamB (Rv0465c) in the control of the glyoxylate cycle in *Mycobacterium tuberculosis.*. J Bacteriol.

[67] Boshoff HI, Myers TG, Copp BR, McNeil MR, Wilson MA (2004). The transcriptional responses of *Mycobacterium tuberculosis* to inhibitors of metabolism: novel insights into drug mechanisms of action.. J Biol Chem.

[68] Trotochaud AE, Wassarman KM (2005). A highly conserved 6S RNA structure is required for regulation of transcription.. Nat Struct Mol Biol.

[69] Panek J, Krasny L, Bobek J, Jezkova E, Korelusova J (2011). The suboptimal structures find the optimal RNAs: homology search for bacterial non-coding RNAs using suboptimal RNA structures.. Nucleic Acids Res.

[70] Keren I, Minami S, Rubin E, Lewis K (2011). Characterization and Transcriptome Analysis of *Mycobacterium tuberculosis* Persisters.. MBio.

[71] Papavinasasundaram KP, Anderson C, Brooks PC, Thomas NA, Movahedzadeh F (2001). Slow induction of RecA by DNA damage in *Mycobacterium tuberculosis*.. Microbiology.

[72] Snapper SB, Melton RE, Mustafa S, Kieser T, Jacobs WRJ (1990). Isolation and characterization of efficient plasmid transformation mutants of *Mycobacterium smegmatis*.. Mol Microbiol.

[73] Croucher NJ, Fookes MC, Perkins TT, Turner DJ, Marguerat SB (2009). A simple method for directional transcriptome sequencing using Illumina technology.. Nucleic Acids Res.

[74] Li H, Handsaker B, Wysoker A, Fennell T, Ruan J (2009). The Sequence Alignment/Map format and SAMtools.. Bioinformatics.

[75] Hunt DM, Saldanha JW, Brennan JF, Benjamin P, Strom M (2008). Single nucleotide polymorphisms that cause structural changes in the cyclic AMP receptor protein transcriptional regulator of the tuberculosis vaccine strain *Mycobacterium bovis* BCG alter global gene expression without attenuating growth.. Infect Immun.

[76] Benjamini Y, Hochberg Y (1995). Controlling the False Discovery Rate: A Practical and Powerful Approach to Multiple Testing.. J R Stat Soc Series B Stat Methodol.

[77] Smoot ME, Ono K, Ruscheinski J, Wang PL, Ideker T (2011). Cytoscape 2.8: new features for data integration and network visualization.. Bioinformatics.

