# Supplementary figures and images for: Sequence-Based Analysis Uncovers an Abundance of Non-Coding RNA in the Total Transcriptome of Mycobacterium tuberculosis
Source: PLoS Pathog. 2011 Nov 3;7(11):e1002342. doi: 10.1371/journal.ppat.1002342 (PMC3207917; doi:10.1371/journal.ppat.1002342)

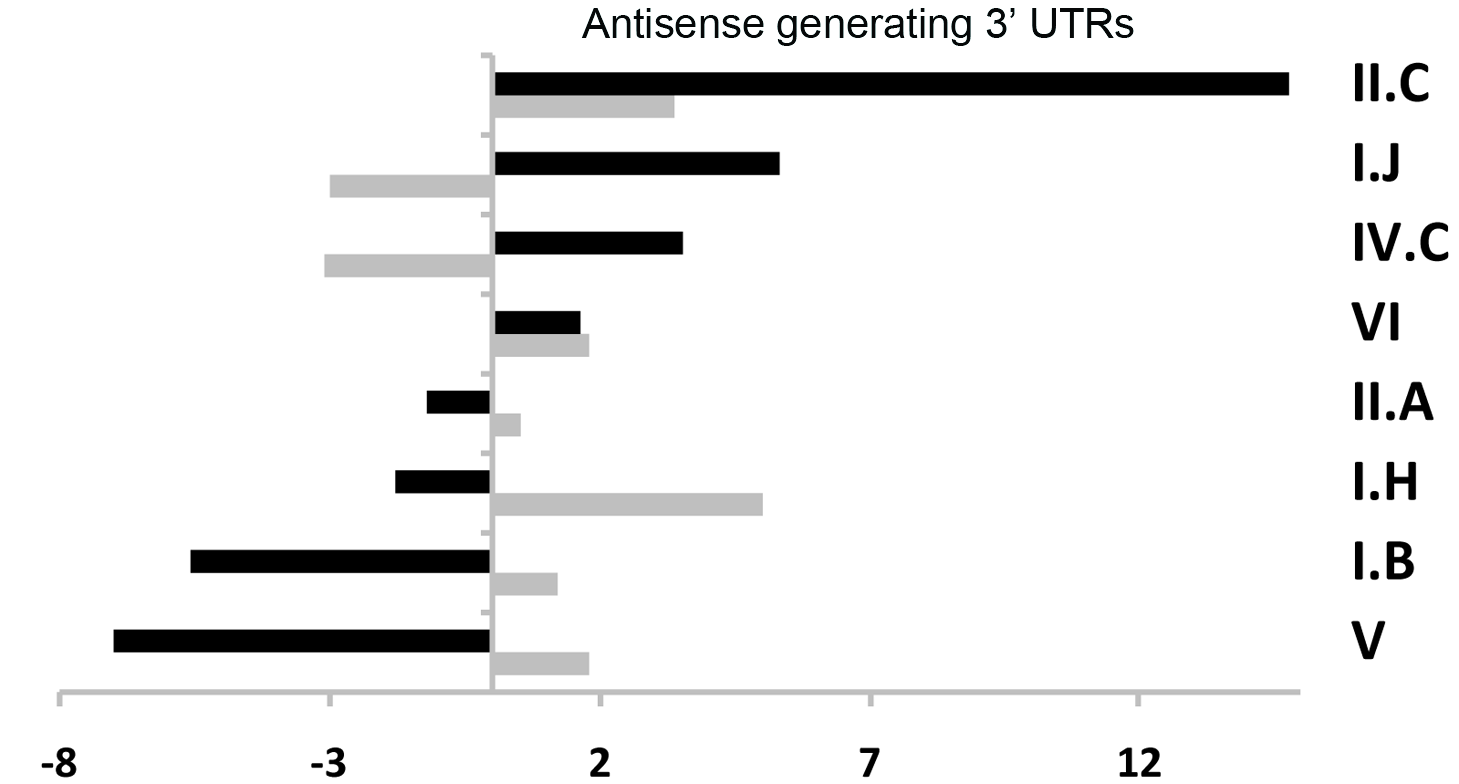

Supplement: Figure S1 — Representation of functional classes amongst genes involved in 3’ UTR antisense overlaps. The difference in frequency of selected functional classes when comparing genes organized in 3’-3’ convergent pairs and having antisense to sense ratio≥0.5 (N = 285) with the total set of CDSs with RPKM≥5 (N = 3,136). Functional class II.C is over-represented (89/245) amongst genes covered by antisense transcripts (Fisher's exact one side test, P−value = 0.034). (TIF) [file ppat.1002342.s001.tif]

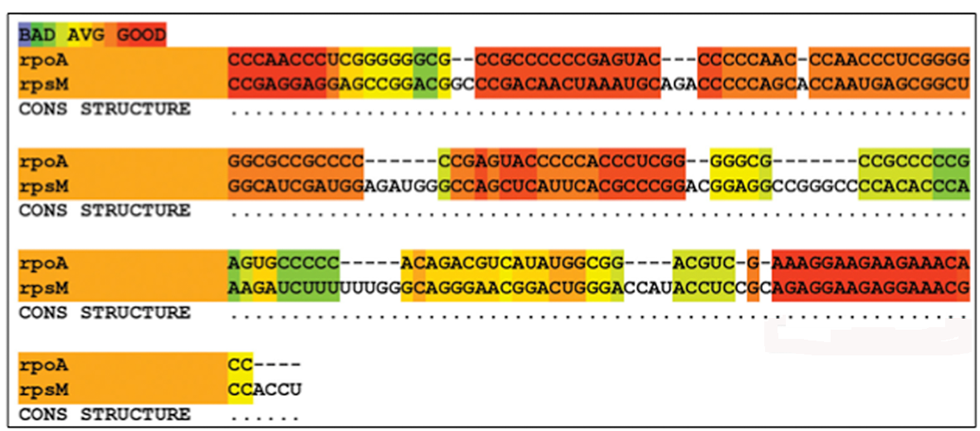

Supplement: Figure S2 — Alignment of long 5’ UTRs from rpoA and rpsM. T-coffee consensus alignment of the 5’ UTRs of rpoA and rpsM (http://genome.ku.dk/resources/war) [40] highlights regions of homology that may play a role in the coordinated regulation of expression. (TIF) [file ppat.1002342.s002.tif]

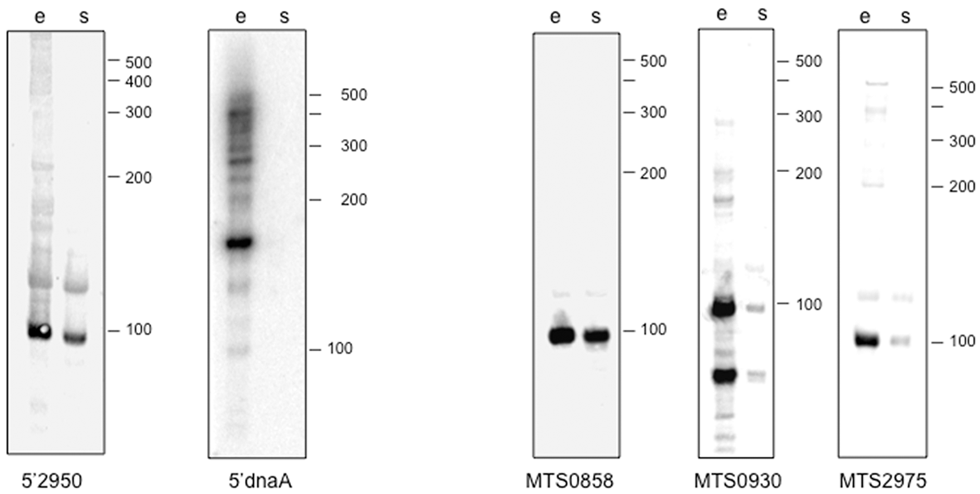

Supplement: Figure S3 — Northern blot confirmation of additional 5’ UTRs and intergenic sRNAs. Each panel shows Northern blots with RNA from exponential (“e”) and stationary (“s”) phase. The stationary phase profile of the 5’ UTR of Rv2950 suggests attenuation. The 5’ UTR of dnaA is not expressed in stationary phase. MTS0858 is prominent in both growth phases, while expression of MTS0930 and MTS2975 is reduced in stationary phase. (TIF) [file ppat.1002342.s003.tif]

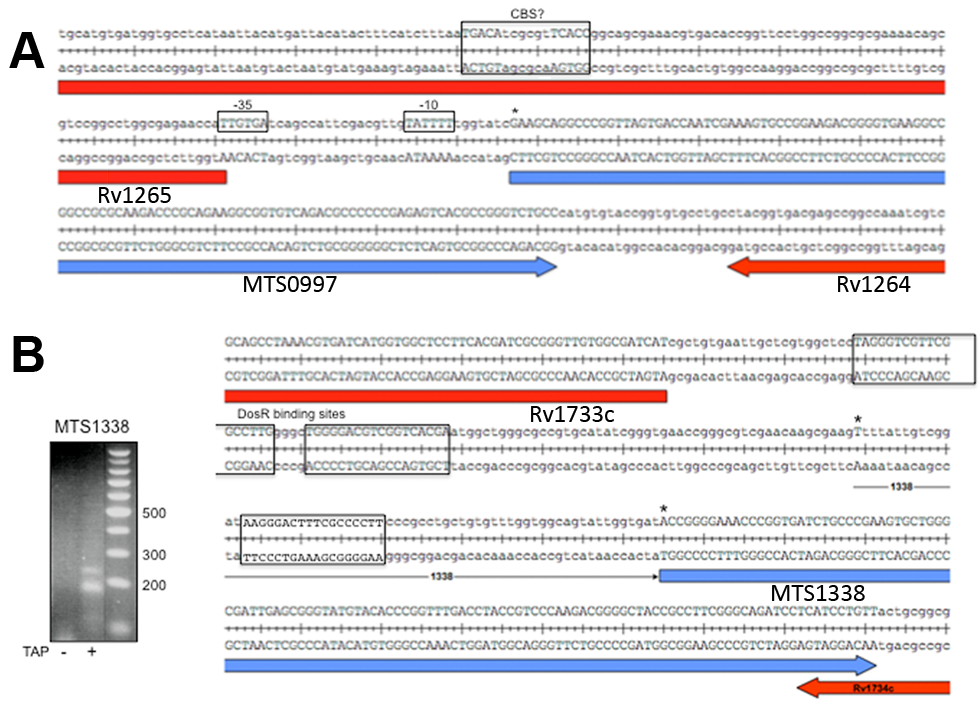

Supplement: Figure S4 — Mapping of sRNA transcriptional start sites and predicted transcription factor binding sites. Panel A shows the region surrounding MTS0977. The 5’ end (not TAP specific) was mapped to the G nucleotide shown by an asterisk. Putative promoter elements as well as CRP binding site (CBS) are outlined by rectangles. The 3’ end is approximate, based on the size of the transcript (as judged by Northern blotting) and structural predictions (using Mfold). The start of Rv1265 was mapped to the −35 element of MTS0997.Panel B shows RLM-RACE result for mapping the transcription start sites of MTS1338 (using primer 1338.R, and indicated by asterisks above the sequence) and the region surrounding MTS1338 with DosR binding sites according to [46] indicated. (TIF) [file ppat.1002342.s004.tif]

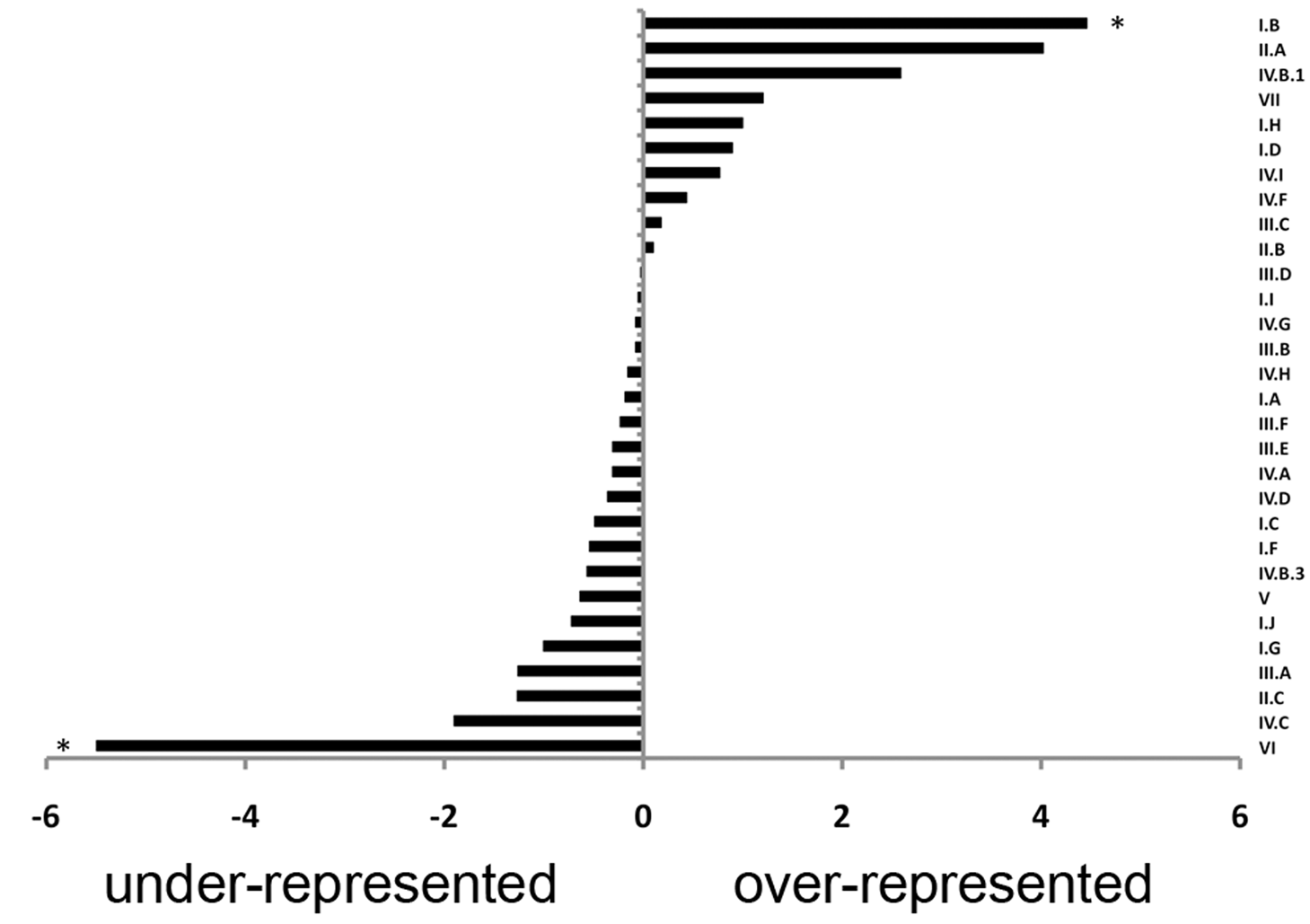

Supplement: Figure S5 — Distribution of down-regulated genes upon over-expression of MTS2823. Genes involved in energy metabolism (class I.B) are over-represented among down-regulated genes. Genes identified as down-regulated ≥2.5-fold by microarray analysis were grouped according to the functional class of their predicted gene product as assigned in the original genome annotation [10]. Values on the x-axis represents a difference in percentage, positive values indicate over-representation of a particular functional class whereas negative values indicate under-representation. Asterisks indicate P−value<0.01 after FDR correction. (TIF) [file ppat.1002342.s005.tif]

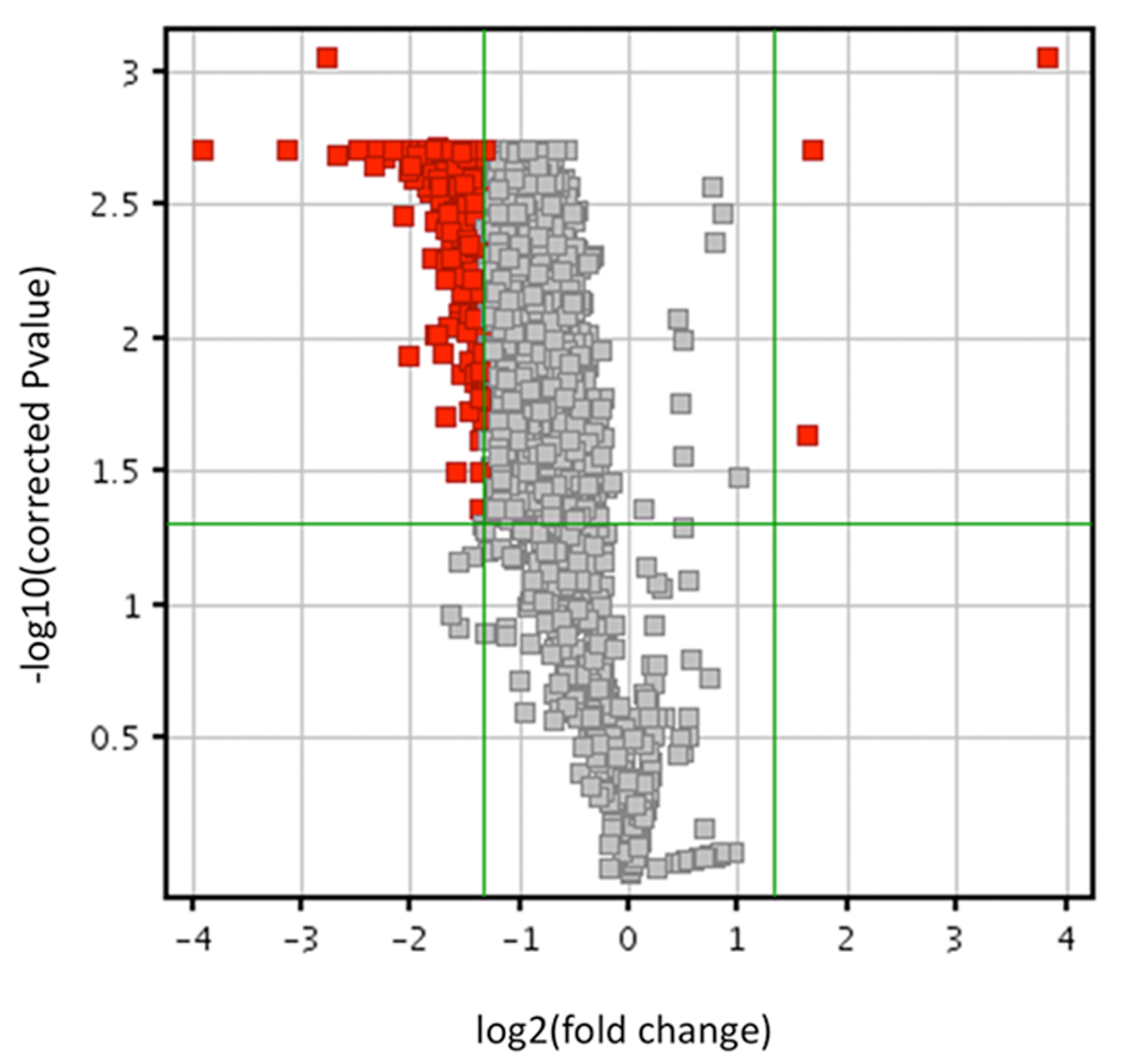

Supplement: Figure S6 — Effect of MTS2823 over-expression on M. tuberculosis genes. The figure shows a Volcano plot of the microarray data, which illustrates how the majority of genes are down-regulated as a result of MTS2823 over-expression. (TIF) [file ppat.1002342.s006.tif]
