# Supplementary material for: Sequence-Based Analysis Uncovers an Abundance of Non-Coding RNA in the Total Transcriptome of Mycobacterium tuberculosis
Source: PLoS Pathog. 2011 Nov 3;7(11):e1002342. doi: 10.1371/journal.ppat.1002342 (PMC3207917; doi:10.1371/journal.ppat.1002342)
Supplement: Table S1 — Pairwise correlation coefficients between five exponential phase samples based on sense and antisense RPKM. (DOC) [file ppat.1002342.s007.doc]

**Table S1: Pairwise correlation coefficient between exponential RPKM (sense + antisense)**

|  | **_4347_8** | **_4349_2** | **_4349_3** | **_4349_5** | **_4349_6** |
| --- | --- | --- | --- | --- | --- |
| **_4347_8** | 1 |  |  |  |  |
| **_4349_2** | 0.939 | 1 |  |  |  |
| **_4349_3** | 0.935 | 0.964 | 1 |  |  |
| **_4349_5** | 0.934 | 0.977 | 0.940 | 1 |  |
| **_4349_6** | 0.930 | 0.951 | 0.967 | 0.955 | 1 |

For all combinations the pvalue=0 (bonferroni corrected)
