# Supplementary material for: Sequence-Based Analysis Uncovers an Abundance of Non-Coding RNA in the Total Transcriptome of Mycobacterium tuberculosis
Source: PLoS Pathog. 2011 Nov 3;7(11):e1002342. doi: 10.1371/journal.ppat.1002342 (PMC3207917; doi:10.1371/journal.ppat.1002342)
Supplement: Table S4 — Expression pattern of predicted riboswitches. (DOC) [file ppat.1002342.s010.doc]

Table S4. Expression pattern of predicted riboswitches

|  |  | |  |  | **RPKM** | | **rbsw/CDS RPKM** | |
| --- | --- | --- | --- | --- | --- | --- | --- | --- |
| **riboswitch** | **genome coordinates** | | **CDS** | **gene** | **rbsw** | **CDS** | **exp** | **sta*** |
| glycine | 79294 | 79095 | Rv0070c | glyA2 | 72 | 23 | 3.1 | 5.8 |
| cobalamin | 309882 | 309685 | Rv0256c | PPE2 | 41 | 22 | 1.9 | (1.5) |
| TPP | 501047 | 501157 | Rv0415 | thiO | 19 | 7 | 2.7 | 7.5 |
| TPP | 510326 | 510216 | Rv0423 | thiC | 22 | 42 | 0.5 | (0.5) |
| ydao-yuaA | 965767 | 965545 | Rv0867c | rpfA | 60 | 130 | 0.5 | (0.9) |
| cobalamin | 1261650 | 1261500 | Rv1133c | metE | 201 | 351 | 0.6 | 0.6 |
| pyr | 1552602 | 1552652 | Rv1379 | pyrR | n/a | n/a | n/a | n/a |
| ykoK(M-box) | 1735509 | 1735678 | Rv1535 | HP | 150 | 163 | 0.9 | 0.7 |
| T-box | 1736287 | 1736518 | Rv1536 | ileS | 147 | 21 | 7.0 | 10.9 |
| M-box | 2047595 | 2047769 | Rv1806 | PE20 | 36 | 9 | 4.0 | 1.0 |
| glycine | 2075613 | 2075824 | Rv1832 | gcvB | 76 | 18 | 4.2 | 13.2 |
| SAM-IV | 3725971 | 3726090 | Rv3340 | metC | 24 | 18 | 1.3 | 3.8 |
| yybP-ykoY | 4322172 | 4322279 | Rv3848 | CMP | 14 | 3 | 4.7 | 10.3 |

n/a: the element was not annotated by the time of mapping

*Values in brackets indicate that RPKMs for 5’ UTR as well as for CDS was lower than 5
